# Biliary excretion of excess iron in mice requires hepatocyte iron import by Slc39a14

**DOI:** 10.1016/j.jbc.2021.100835

**Published:** 2021-05-26

**Authors:** Milankumar Prajapati, Heather L. Conboy, Shintaro Hojyo, Toshiyuki Fukada, Bogdan Budnik, Thomas B. Bartnikas

**Affiliations:** 1 Department of Pathology and Laboratory Medicine, Brown University, Providence, Rhode Island, USA; 2 Division of Molecular Psychoimmunology, Institute for Genetic Medicine, Graduate School of Medicine, Hokkaido University, Hokkaido, Japan; 3 Department of Molecular and Cellular Physiology, Faculty of Pharmaceutical Sciences, Tokushima Bunri University, Tokushima, Japan; 4 Mass Spectrometry and Proteomics Resource Laboratory, Faculty of Arts and Sciences, Division of Science, Harvard University, Cambridge, Massachusetts, USA

**Keywords:** iron, iron metabolism, bile, liver, transport, hepatocyte, SLC39A14, ferritin, Fe, iron, Fth1, ferritin heavy chain, Ftl1, Ferritin light chain, Hamp, hepcidin, Ltf, lactoferrin, Mn, manganese, NTBI, non-transferrin-bound iron, Tf, transferrin

## Abstract

Iron is essential for erythropoiesis and other biological processes, but is toxic in excess. Dietary absorption of iron is a highly regulated process and is a major determinant of body iron levels. Iron excretion, however, is considered a passive, unregulated process, and the underlying pathways are unknown. Here we investigated the role of metal transporters SLC39A14 and SLC30A10 in biliary iron excretion. While SLC39A14 imports manganese into the liver and other organs under physiological conditions, it imports iron under conditions of iron excess. SLC30A10 exports manganese from hepatocytes into the bile. We hypothesized that biliary excretion of excess iron would be impaired by SLC39A14 and SLC30A10 deficiency. We therefore analyzed biliary iron excretion in *Slc39a14*-and *Slc30a10*-deficient mice raised on iron-sufficient and -rich diets. Bile was collected surgically from the mice, then analyzed with nonheme iron assays, mass spectrometry, ELISAs, and an electrophoretic assay for iron-loaded ferritin. Our results support a model in which biliary excretion of excess iron requires iron import into hepatocytes by SLC39A14, followed by iron export into the bile predominantly as ferritin, with iron export occurring independently of SLC30A10. To our knowledge, this is the first report of a molecular determinant of mammalian iron excretion and can serve as basis for future investigations into mechanisms of iron excretion and relevance to iron homeostasis.

Iron (Fe) is a dietary nutrient essential for erythropoiesis and other biological processes (1). In excess, Fe catalyzes the formation of reactive oxygen species, leading to dysfunction in organs such as the liver, heart, and pancreas. Fe absorption is a critical determinant of body Fe levels. Much of our understanding of Fe absorption comes from the study of inherited diseases of Fe excess such as hereditary hemochromatosis (2). Hereditary hemochromatosis is caused by deficiency in hepcidin, a hormone that inhibits dietary Fe absorption. Hepcidin is synthesized mainly by the liver. Its expression is stimulated by Fe and inflammation and inhibited by anemia. Hepcidin inhibits Fe absorption by posttranslationally downregulating expression and activity of ferroportin, a protein that exports Fe from enterocytes and macrophages into the blood. In hereditary hemochromatosis, hepcidin deficiency leads to unabated Fe absorption, Fe excess, and toxicity.

In theory, Fe levels are determined by absorption and excretion. Our understanding of mechanisms of Fe absorption has advanced greatly over recent decades. In contrast, little is established about pathways of Fe excretion. Excretion is considered passive and unregulated. It is attributed to turnover of intestinal epithelium, blood loss, and exfoliation of dead skin (1). However, Fe can also undergo biliary excretion (3). Some studies report that only minor amounts of Fe are excreted in the bile under conditions of Fe excess, while others report that increases in bile Fe levels are not proportionate to increases in liver Fe levels in conditions of Fe excess (4, 5, 6, 7, 8). Nevertheless, non-transferrin-bound Fe (NTBI), a pathologic form of Fe present in states of Fe excess, is excreted into the bile (9, 10). NTBI also undergoes enterohepatic circulation, the process by which substances in the bile are reabsorbed by the intestines for return to the liver. Inorganic Fe can bind to bile salts and remains soluble at neutral pH (11, 12). Fe-binding proteins are also present in the bile. Transferrin and lactoferrin translocate from the blood to the bile (13, 14, 15). Ferritin is present in the bile and originates from lysosomal exocytosis by hepatocytes (16, 17, 18, 19, 20, 21). However, only transferrin is abundant in the bile under physiologic conditions (22).

To explore the molecular basis of biliary Fe excretion, we focused on the metal transporter SLC39A14 (ZIP14). Under physiologic conditions, SLC39A14 imports manganese (Mn) from the blood into hepatocytes and enterocytes (23, 24, 25, 26, 27). Patients with SLC39A14 deficiency develop a rare inherited disease of Mn excess and neurologic dysfunction due to impaired gastrointestinal Mn excretion (28, 29). However, under conditions of Fe excess, SLC39A14 imports NTBI into hepatocytes and pancreatic acinar cells (30, 31). Slc39a14 deficiency attenuates liver Fe excess in mice on Fe-rich diets or in mice deficient in hemochromatosis proteins Hfe or Hfe2 or hepatic transferrin. To our knowledge, SLC39A14 is the only known transporter shown *in vivo* to be specific to NTBI. For this reason, and because SLC39A14 also imports NTBI into hepatocytes, we focused on this transporter in this study on biliary excretion of excess Fe. Specifically, we hypothesized that biliary excretion of excess Fe would be impaired by SLC39A14 deficiency because import of excess Fe into hepatocytes is impaired by SLC39A14 deficiency. To test this, we analyzed biliary Fe excretion in Slc39a14-deficient mice (*Slc39a14*^*KO/KO*^) raised on Fe-sufficient and -rich diets.

We also studied the metal transporter SLC30A10 (ZNT10). SLC30A10 exports Mn from hepatocytes into the bile and from enterocytes into the lumen of the gastrointestinal tract (32, 33, 34, 35, 36). Like SLC39A14 deficiency, SLC30A10 deficiency causes a rare inherited disease of Mn excess and neurological dysfunction due to impaired gastrointestinal Mn excretion (28, 29). (In contrast, unlike SLC39A14 deficiency, SLC30A10 deficiency also leads to liver cirrhosis and polycythemia. While the cause of polycythemia is unclear, liver cirrhosis is attributed to liver Mn excess.) As mentioned above, SLC39A14 imports Mn and NTBI into the liver. Given that SLC3010 exports Mn from the liver, we considered that SLC30A10 may also export NTBI from the liver and that biliary excretion of excess Fe may be impaired by SLC30A10 deficiency. To test this, we analyzed biliary Fe excretion in Slc30a10-deficient mice (*Slc30a10*^*KO/KO*^) raised on Fe-sufficient and -rich diets.

Here we present our analysis of SLC39A14 and SLC30A10 in biliary Fe excretion and discuss the implications of our findings to the understanding of Fe homeostasis.

## Results

### Slc39a14 deficiency impairs liver Fe loading and biliary Fe excretion in mice on Fe-rich diet

As described above, we hypothesized that biliary excretion of excess Fe by hepatocytes would be impaired by Slc39a14 deficiency, because import of excess Fe into hepatocytes requires Slc39a14. To test this, we first established that Slc39a14 deficiency impairs hepatocyte Fe loading in mice with dietary Fe loading, as previously reported (30). *Slc39a14*^*+/+*^ and *Slc39a14*^*KO/KO*^ mice were weaned onto diets containing 50 or 10,000 ppm Fe. Bile, blood, and tissues were then collected from mice at 2 months of age and first analyzed for phenotypes relevant to Fe homeostasis.

The Fe-rich diet had no effect on body mass, although male *Slc39a14*^*KO/KO*^ mice were smaller than *Slc39a14*^*+/+*^ mice raised on either diet (Fig. S1*A*). The Fe-rich diet also had no impact on hemoglobin levels, hematocrits, serum Fe levels, or transferrin saturations in *Slc39a14*^*+/+*^ or *Slc39a14*^*KO/KO*^ mice (Fig. S1, *B*–*E*). In contrast, the Fe-rich diet increased liver mass in all mice (Fig. S1*F*). It also increased nonheme Fe levels in all mice although liver Fe levels were lower in *Slc39a14*^*KO/KO*^ than *Slc39a14*^*+/+*^ mice raised on Fe-rich diets (Fig. S1*G*). The Fe-rich diet increased hepcidin RNA levels in all mice although the increase in female *Slc39a14*^*+/+*^ mice did not reach significance (Fig. S1*H*). Fe levels were also analyzed in other tissues. The Fe-rich diet increased Fe levels in the spleens of *Slc39a14*^*KO/KO*^ mice (Fig. S1*I*). It also increased Fe levels in the kidney and heart of *Slc39a14*^*KO/KO*^ but not *Slc39a14*^*+/+*^ mice (Fig. S1, *J* and *K*), but had no effect on Fe levels in the pancreas for any mice (Fig. S1*L*).

We next performed tissue Fe stains to examine the cellular localization of excess Fe. Fe was detected mainly in periportal hepatocytes in *Slc39a14*^*+/+*^ mice and in nonparenchymal cells in *Slc39a14*^*KO/KO*^ mice raised on Fe-rich diets (Fig. S1, *M* and *N*). Fe staining was not observed in the pancreas for any mouse group (Fig. S2*A*). Fe was detected in splenic red pulp macrophages, with staining most intense in *Slc39a14*^*KO/KO*^ mice raised on Fe-rich diets (Fig. S2*B*). (Fe staining was undetectable in the spleens of male *Slc39a14*^*KO/KO*^ mice raised on the Fe-sufficient diet. A similar finding was noted with nonheme Fe measurements (Fig. S1*I*). The relevance of this is not clear.) Fe was detected in enterocytes of mice on Fe-rich diets, with staining most prominent near the apical surface, although intensity varied regionally even within samples (Fig. S2, *C* and *D*).

**Figure 1:**
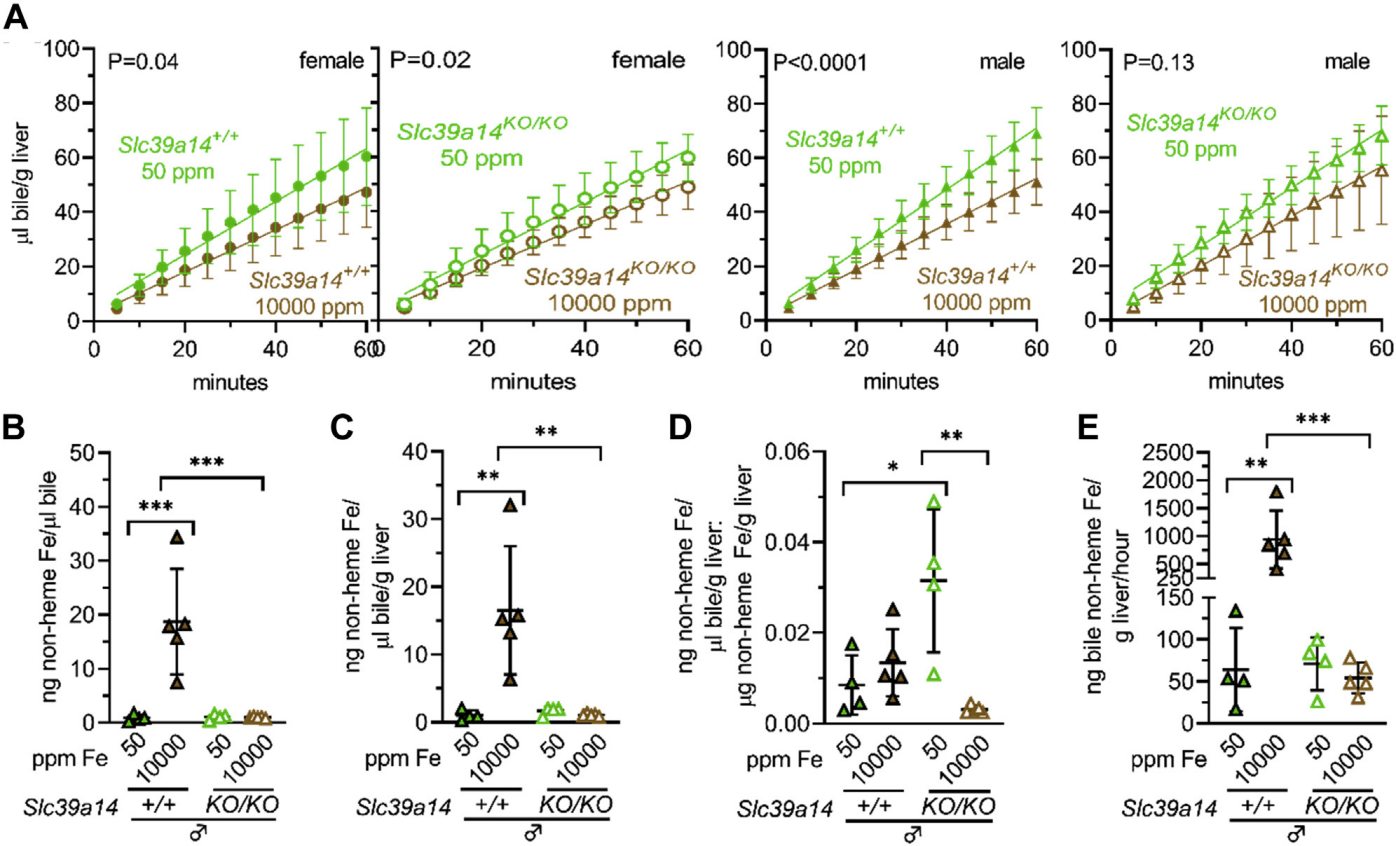
**Slc39a14 deficiency impairs biliary excretion of excess Fe.***Slc39a14*^*+/+*^ and *Slc39a14*^*KO/KO*^ mice were weaned onto Fe-sufficient or -rich diets then underwent bile, blood, and tissue collection at 2 months old. *A*, bile flow rates; curves compared by linear regression. *B* and *C*, bile nonheme Fe levels without (*B*) and with (*C*) normalization to liver mass. *D*, ratios of bile to liver nonheme Fe levels. *E*, bile nonheme Fe levels with normalization to liver mass, excreted per hour. Bars indicate mean ± standard deviation. For (*B*–*E*), groups within each sex compared by one-way ANOVA with Tukey’s post-hoc test (ns *p* ≥ 0.05, ∗*p* < 0.05, ∗∗*p* < 0.01, ∗∗∗*p* < 0.001, ∗∗∗∗*p* < 0.0001).

The above analyses indicated that Slc39a14 deficiency impaired hepatocyte Fe loading in mice raised on an Fe-rich diet, as previously reported (30). To test the hypothesis that biliary excretion of excess Fe is impaired by Slc39a14 deficiency, we next analyzed the bile collected surgically from mice at the time of harvest. The Fe-rich diet decreased bile flow rates in all mice except male *Slc39a14*^*KO/KO*^ mice (Fig. 1*A*). Bile flow rates did not differ between *Slc39a14*^*+/+*^ and *Slc39a14*^*KO/KO*^ mice for either sex on either diet (*p* values not shown in Fig. 1*A*). The Fe-rich diet increased bile nonheme Fe levels in *Slc39a14*^*+/+*^ mice but not in *Slc39a14*^*KO/KO*^ mice, with and without normalization to liver mass (Fig. 1, *B* and *C*). To determine if increases in bile Fe levels were proportionate to increases in liver Fe levels, we calculated ratios of bile to liver Fe levels. The Fe-rich diet had no impact on ratios in *Slc39a14*^*+/+*^ mice but did decrease them in *Slc39a14*^*KO/KO*^ mice (Fig. 1*D*). The Fe-rich diet also increased nonheme Fe levels excreted into the bile per hour in *Slc39a14*^*KO/KO*^ mice but not *Slc39a14*^*KO/KO*^ mice (Fig. 1*E*). Overall, these data indicated that Slc39a14 deficiency impairs biliary excretion of excess Fe under conditions of dietary Fe loading. (There was insufficient female mouse bile for all analyses in this report. Bile yields were lower for females given their smaller size. Also, female bile samples were analyzed by mass spectrometry, as described below.)

### Slc39a14 deficiency impairs biliary excretion of Fe-rich ferritin in mice on Fe-rich diet

**Figure 2:**
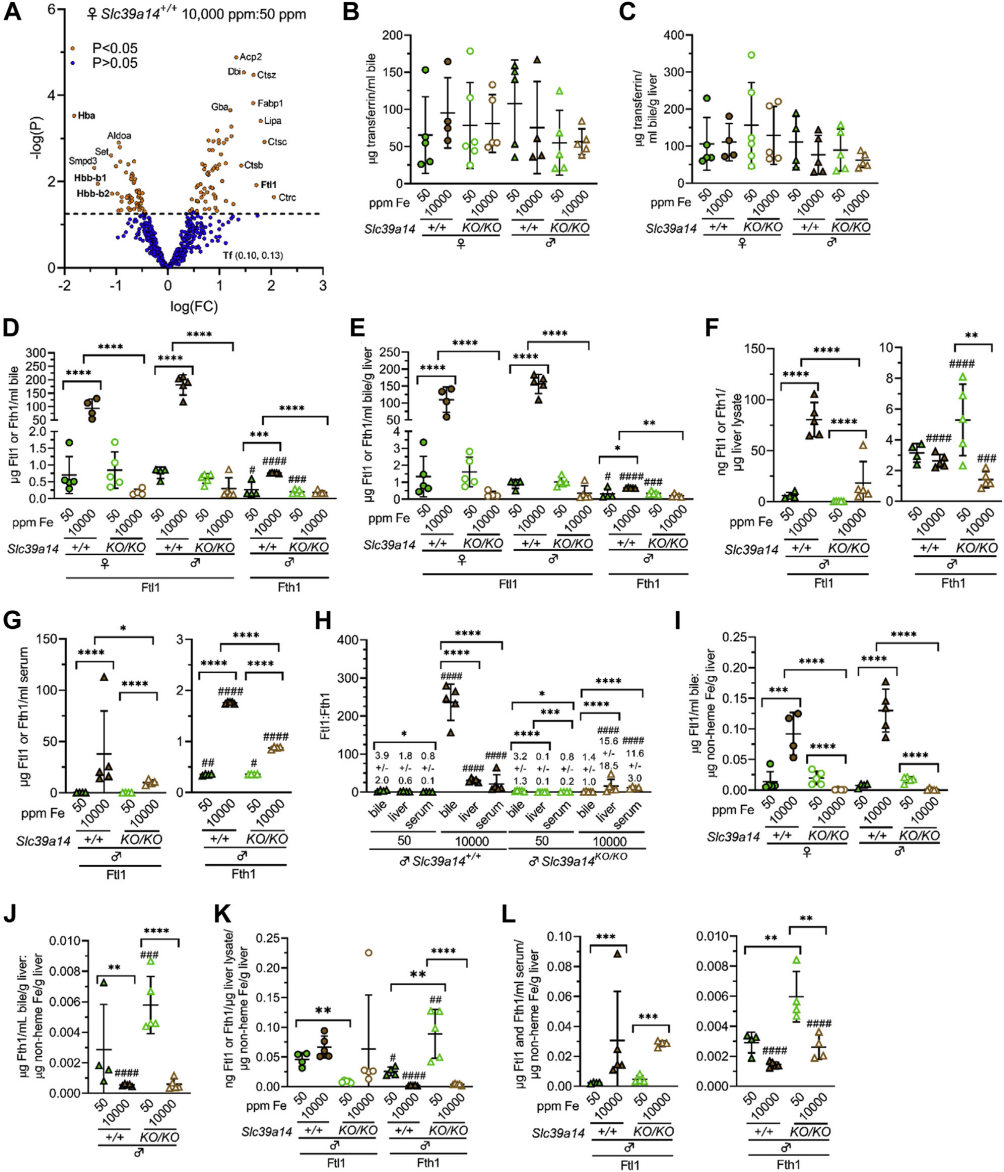
**Slc39a14 deficiency impairs biliary excretion of ferritin in mice on Fe-rich diet.***A*, volcano plot of relative abundance of bile proteins in female *Slc39a14*^*+/+*^ mice on Fe-rich (n = 5) and –sufficient (n = 5) diets as measured by mass spectrometry. *Dashed line* indicates *p* = 0.05; *orange* and *blue circles* indicate points with *p* < 0.05 and *p* > 0.05 respectively. Proteins with relevance to Fe or heme homeostasis in *bold*. Numbers adjacent to Tf indicate log(FC) and –log(*p*) values. Proteins with single-peptide identification were excluded. *B*–*G*, protein levels in bile, liver lysates, and serum from *Slc39a14*^*+/+*^ and *Slc39a14*^*KO/KO*^ mice on Fe-sufficient and -rich diets as measured by ELISA. *B* and *C*, bile Tf levels without (*B*) and with (*C*) normalization to liver mass. *D* and *E*, bile Ftl1 and Fth1 levels without (*D*) and with (*E*) normalization to liver mass. *F*, liver lysate Ftl1 and Fth1 levels. *G*, serum Ftl1 and Fth1 levels. *H*, ratios of Ftl1 to Fth1 levels. *I*–*L*, ratios of bile (*I* and *J*), liver lysate (*K*), and serum (*L*) Ftl1 and Fth1 levels to liver nonheme Fe levels. For (*B*–*G*, *I*–*L*), groups within each sex compared by one-way ANOVA with Tukey’s post-hoc test as in Figure 1. Ftl1 and Fth1 levels within each group (*B*–*G*, *I*–*L*) and Ftl1:Fth1 ratios between samples from Fe-sufficient and -rich diets (*H*) compared by unpaired, two-tailed *t* test (#*p* < 0.05, ##*p* < 0.01, ###*p* < 0.001, ####*p* < 0.0001). Bars indicate mean ± standard deviation.

To determine the biochemical nature of excess Fe in the bile, we first identified proteins abundantly expressed in the bile from Fe-loaded mice. Bile samples from five female *Slc39a14*^*+/+*^ mice raised on Fe-sufficient and -rich diets were digested with trypsin, labeled with TMT11plex, fractionated, and analyzed by LC-MS/MS (Fig. 2*A*, Tables 1 and 2). Hemoglobin subunits Hba, Hbb-b1, and Hbb-b2 were more abundant in mice on the Fe-sufficient diet. Ferritin light chain (Ftl1) was more abundant in mice on the Fe-rich diet. Ferritin heavy chain (Fth1), lactoferrin (Ltf), and transferrin (Tf) did not differ in abundance. (Both Fth1 and Ltf were identified only by single unique peptides.)

**Table 1:** Bile proteins identified by mass spectrometry as less abundant (*p* < 0.05) in bile from female *Slc39a14*^*+/+*^ mice raised on Fe-rich diet than on Fe-sufficient diet, arranged in descending order by –log(*p*)

Symbol	Name	Accession	%	#	FC	logFC	*p*	adj *p*	−log *p*
Hba	Hemoglobin subunit alpha	P01942	31	6	−3.45	−1.82	2.97E-04	2.38E-02	3.53
Aldoa	Fructose-bisphosphate aldolase A	P05064	16	7	−1.80	−0.95	1.25E-03	4.40E-02	2.90
Hist1h1c	Histone H1.2	P15864	31	3	−1.77	−0.93	1.59E-03	4.93E-02	2.80
Set	Protein SET	Q9EQU5	5	2	−2.02	−1.10	2.47E-03	6.23E-02	2.61
Ca2	Carbonic anhydrase 2	P00920	7	2	−1.76	−0.92	3.43E-03	7.49E-02	2.46
Sept8	Septin-8	Q8CHH9	4	2	−1.88	−1.00	4.40E-03	7.97E-02	2.36
Smpd3	Sphingomyelin phosphodiesterase 3	Q9JJY3	6	2	−2.59	−1.42	4.80E-03	8.15E-02	2.32
Nars	Asparagine--tRNA ligase, cytoplasmic	Q8BP47	3	2	−1.61	−0.81	6.13E-03	8.92E-02	2.21
Hbb-b1	Hemoglobin subunit beta-1	P02088	65	5	−2.46	−1.36	1.12E-02	1.21E-01	1.95
Calm2	Calmodulin-2	P0DP27	13	3	−1.84	−0.98	1.89E-02	1.54E-01	1.72
Hbb-b2	Hemoglobin subunit beta-2	P02089	47	5	−2.00	−1.08	1.91E-02	1.54E-01	1.72
Ppt1	Palmitoyl-protein thioesterase 1	O88531	17	7	−1.72	−0.89	2.28E-02	1.64E-01	1.64
Acot1	Acyl-coenzyme A thioesterase 1	O55137	6	3	−1.73	−0.90	2.90E-02	1.81E-01	1.54
Uqcrc1	Cytochrome b-c1 complex subunit 1, mitochondrial	Q9CZ13	5	3	−1.74	−0.90	4.55E-02	2.34E-01	1.34
Atp5f1b	ATP synthase subunit beta, mitochondrial	P56480	28	14	−1.81	−0.96	4.68E-02	2.38E-01	1.33

Accession: accession number from UniProtKB; %, percent coverage; #, number unique peptides; adj *p*, adjusted *p*. Proteins with single-peptide identification excluded.

**Table 2:** Bile proteins identified by mass spectrometry as more abundant (*p* < 0.05) in bile from female *Slc39a14*^*+/+*^ mice raised on Fe-rich diet than on Fe-sufficient diet, arranged in descending order by –log(*p*)

Symbol	Name	Accession	%	#	FC	logFC	*p*	adj.*p*	−log *p*
Acp2	Lysosomal acid phosphatase	P24638	5	3	2.40	1.32	1.31E-05	3.86E-03	4.88
Dbi	Acyl-CoA-binding protein	P31786	15	2	2.68	1.47	2.94E-05	5.18E-03	4.53
Ctsz	Cathepsin Z	Q9WUU7	9	3	3.08	1.66	3.34E-05	5.18E-03	4.48
Fabp1	Fatty acid-binding protein, liver	P12710	38	7	3.06	1.65	1.51E-04	1.50E-02	3.82
Gba	Glucosylceramidase	P17439	5	3	2.20	1.21	2.20E-04	1.92E-02	3.66
Lipa	Lysosomal acid lipase/cholesteryl ester hydrolase	Q9Z0M5	4	2	3.39	1.79	3.91E-04	2.68E-02	3.41
Gm2a	Ganglioside GM2 activator	Q60648	24	4	2.23	1.23	5.33E-04	3.20E-02	3.27
Enpep	Glutamyl aminopeptidase	P16406	8	8	1.81	0.96	6.53E-04	3.69E-02	3.19
Reg3b	Regenerating islet-derived protein 3	P35230	8	2	2.24	1.23	8.78E-04	4.40E-02	3.06
Tpm4	Tropomyosin alpha-4 chain	Q6IRU2	12	3	2.08	1.14	1.05E-03	4.40E-02	2.98
Scp2	Non-specific lipid-transfer protein	P32020	5	3	1.85	0.99	1.10E-03	4.40E-02	2.96
Gaa	Lysosomal alpha-glucosidase	P70699	7	7	1.70	0.88	1.16E-03	4.40E-02	2.94
Ctsc	Dipeptidyl peptidase 1	P97821	5	3	3.58	1.87	1.20E-03	4.40E-02	2.92
Plbd2	Putative phospholipase B-like	Q3TCN2	6	4	2.00	1.08	1.26E-03	4.40E-02	2.90
Man2b1	Lysosomal alpha-mannosidase	O09159	2	2	1.85	0.98	1.28E-03	4.40E-02	2.89
Gns	N-acetylglucosamine-6-sulfatase	Q8BFR4	4	2	1.60	0.80	1.84E-03	5.28E-02	2.74
Cat	Catalase	P24270	13	8	1.68	0.87	1.87E-03	5.28E-02	2.73
Fabp5	Fatty acid-binding protein, epidermal	Q05816	9	2	1.91	1.03	2.31E-03	6.20E-02	2.64
Decr2	Peroxisomal 2,4-dienoyl-CoA reductase	Q9WV68	4	2	2.05	1.12	3.74E-03	7.97E-02	2.43
Hsd17b4	Peroxisomal multifunctional enzyme type 2	P51660	3	2	1.87	0.99	4.02E-03	7.97E-02	2.40
Ctsb	Cathepsin B	P10605	14	4	2.58	1.42	4.25E-03	7.97E-02	2.37
Ctsh	Pro-cathepsin H	P49935	7	4	1.76	0.92	4.40E-03	7.97E-02	2.36
Pbld1	Phenazine biosynthesis-like domain-containing protein	Q9DCG6	9	2	1.71	0.89	4.93E-03	8.15E-02	2.31
Smpdl3a	Acid sphingomyelinase-like phosphodiesterase 3a	P70158	4	2	1.81	0.96	4.94E-03	8.15E-02	2.31
Man2b2	Epididymis-specific alpha-mannosidase	O54782	3	5	1.68	0.86	5.45E-03	8.31E-02	2.26
Gsta3	Glutathione S-transferase A3	P30115	25	6	1.95	1.05	9.73E-03	1.13E-01	2.01
Ftl1	Ferritin light chain 1	P29391	32	7	3.19	1.71	1.21E-02	1.23E-01	1.92
Adh5	Alcohol dehydrogenase class-3	P28474	8	4	1.63	0.83	1.37E-02	1.30E-01	1.86
Crot	Peroxisomal carnitine O-octanoyltransferase	Q9DC50	3	2	1.67	0.86	1.39E-02	1.30E-01	1.86
Cpq	Carboxypeptidase Q	Q9WVJ3	7	4	1.68	0.86	1.42E-02	1.31E-01	1.85
Adh1	Alcohol dehydrogenase 1	P00329	5	2	1.72	0.89	1.99E-02	1.56E-01	1.70
Iap	Intestinal-type alkaline phosphatase	P24822	3	2	1.75	0.92	2.20E-02	1.63E-01	1.66
Ctrc	Chymotrypsin-C	Q3SYP2	5	2	4.08	2.05	2.28E-02	1.64E-01	1.64
Rgn	Regucalcin	Q64374	13	4	1.76	0.92	2.44E-02	1.65E-01	1.61
Ctsd	Cathepsin D	P18242	16	8	2.06	1.12	2.82E-02	1.78E-01	1.55
Acad11	Acyl-CoA dehydrogenase family member 11	Q80XL6	2	2	2.30	1.27	3.33E-02	1.94E-01	1.48
Sod1	Superoxide dismutase [Cu-Zn]	P08228	27	6	1.75	0.91	4.03E-02	2.23E-01	1.40
Serpina3k	Serine protease inhibitor A3K	P07759	42	16	1.80	0.95	4.73E-02	2.38E-01	1.33

Accession: accession number from UniProtKB; %, percent coverage; #, number unique peptides; adj *p*, adjusted *p*. Proteins with single-peptide identification excluded.

Tf was readily detected in our bile analysis. Given this, we considered that increased Fe saturation of bile Tf could contribute to increased bile nonheme Fe levels, even though mass spectrometry indicated that Tf did not differ in abundance between bile samples from mice on Fe-sufficient and -rich diets. By ELISA, Tf levels were ∼50 to 100 μg/ml bile (and ∼50–150 μg/ml bile/g liver) across all groups (Fig. 2, *B* and *C*). Assuming a molecular weight of 80 kD for Tf, 50 to 100 μg Tf/ml equates to 0.625 to 1.25 μM, which could bind 1.25 to 2.5 μM Fe or 70 to 140 ng Fe/ml bile (Table S1). For reference, bile nonheme Fe levels were ∼20 μg/ml in *Slc39a14*^*+/+*^ mice on Fe-rich diets (Fig. 1*B*). This suggested that the majority of bile nonheme Fe was not bound to Tf.

Since Ftl1 levels were more abundant in mice on the Fe-rich diet, we next focused on ferritin subunits Ftl1 and Fth1. Bile Ftl1 and Fth1 levels were measured by ELISA. The Fe-rich diet increased bile Ftl1 and Fth1 levels in *Slc39a14*^*+/+*^ but not *Slc39a14*^*KO/KO*^ mice, although Fth1 levels were lower than Ftl1 levels for all mice except *Slc39a14*^*KO/KO*^ mice on Fe-rich diets (Fig. 2*D*). Similar results were observed when bile Ftl1 and Fth1 levels were normalized to liver mass (Fig. 2*E*). We next measured Ftl1 and Fth1 in liver lysates and serum by ELISA for comparison. We first analyzed the liver. The Fe-rich diet increased liver lysate Ftl1 levels in *Slc39a14*^*+/+*^ and *Slc39a14*^*KO/KO*^ mice, although levels were lower in *Slc39a14*^*KO/KO*^ mice than *Slc39a14*^*+/+*^ mice raised on the Fe-rich diet (Fig. 2*F*, left graph). In contrast, the Fe-rich diet decreased liver lysate Fth1 levels but only in *Slc39a14*^*KO/KO*^ mice (Fig. 2*F*, right graph). Ftl1 levels were higher than Fth1 levels for all groups except *Slc39a14*^*+/+*^ mice raised on the Fe-sufficient diet. We next focused on the serum. The Fe-rich diet increased serum Ftl1 and Fth1 levels in *Slc39a14*^*+/+*^ and *Slc39a14*^*KO/KO*^ mice, although Ftl1 levels were lower in *Slc39a14*^*KO/KO*^ than *Slc39a14*^*+/+*^ mice raised on Fe-rich diets and Fth1 levels were lower than Ftl1 levels for all groups (Fig. 2*G*). To compare the relative abundance of ferritin subunits in the bile, liver lysates, and serum, we next calculated ratios of Ftl1 to Fth1 levels. Ftl1:Fth1 ratios were greater in the bile than liver or serum in *Slc39a14*^*+/+*^ mice on Fe-rich diets but lower in the bile than liver or serum in *Slc39a14*^*KO/KO*^ mice on Fe-rich diets (Fig. 2*H*). The Fe-rich diet increased Ftl1:Fth1 ratios for all compartments for all mice except *Slc39a14*^*KO/KO*^ bile. Overall, these data confirmed the mass spectrometric analysis indicating that ferritin is enriched in Fe-loaded bile. The difference in Ftl1:Fth1 ratios for the bile, liver lysates, and serum also suggested that bile ferritin is biochemically distinct from liver and serum ferritin in conditions of dietary Fe loading.

We next evaluated if Ftl1 and Fth1 levels were proportionate to liver Fe levels by calculating ratios of Ftl1 or Fth1 to liver nonheme Fe levels. For bile, the Fe-rich diet increased Ftl1:liver Fe ratios in *Slc39a14*^*+/+*^ mice but decreased ratios in *Slc39a14*^*KO/KO*^ mice (Fig. 2*I*), while the Fe-rich diet decreased bile Fth1:liver Fe ratios in all mice (Fig. 2*J*). For liver lysates, the Fe-rich diet decreased Fth1:liver Fe ratios in male *Slc39a14*^*KO/KO*^ mice (Fig. 2*K*). For serum, the Fe-rich diet increased Ftl1:liver Fe ratios in all mice and decreased Fth1:liver Fe ratios in male *Slc39a14*^*KO/KO*^ mice (Fig. 2*L*).

To determine if bile ferritin levels were sufficiently elevated in Fe-rich bile to account for all nonheme Fe, we next performed several analyses. First, we estimated the amount of Fe that could be bound to ferritin in bile of Fe-loaded mice. Bile nonheme Fe and Ftl1 levels in *Slc39a14*^*+/+*^ mice on Fe-rich diets were ∼20 and ∼175 μg/ml respectively (Figs. 1*B* and 2D), which equated to ∼350 and ∼8 μM assuming a molecular weight of 21 kD for Ftl1 (Table S1). If bile ferritin consists of 24 subunits and all nonheme Fe was ferritin-bound, each bile holo-ferritin would need to bind ∼1000 Fe atoms. Ferritins can bind up to 4000 atoms of Fe (37). This calculation suggested that bile ferritin levels were sufficient to bind all nonheme Fe present in the bile from *Slc39a14*^*+/+*^ mice raised on the Fe-rich diet.

To more directly assess Fe loading of bile, we employed a gel electrophoresis approach to detect Fe-loaded ferritin. The same Fe stain used for tissue histology in Figs. S1 and S2 detects a slowly migrating species in Fe-rich liver lysates run on native PAGE; this species comigrates with Ftl1 and Fth1 in native immunoblots (38). We reproduced that finding here using native PAGE, Fe staining, and Ftl1 and Fth1 immunoblots of liver lysates from *Slc39a14*^*+/+*^ mice raised on Fe-sufficient and -rich diets (Fig. S3*A*). To determine if the Fe-stainable band was ferritin, we adapted a methanol precipitation/heat denaturation protocol that enriches for ferritin in liver lysates (39). Liver lysates from *Slc39a14*^*+/+*^ mice on Fe-sufficient or -rich diets were pooled, then treated with methanol, heated, and centrifuged. Supernatants were separated into filtrates and retentates using centrifugal filters with a 100 kD molecular weight cutoff. Most liver proteins precipitated after methanol/heat treatment (Fig. S3*B*, top image, lanes 3 and 4 *versus* 1 and 2). There were minimal proteins detected in filtrates (Fig. S3*B*, top image, lanes 5 and 6). There were few proteins found in retentates, with one species of ∼25 kD more abundant in Fe-rich liver lysates (Fig. S3*B*, top image, lanes 7 and 8, arrow). Ftl1 and Fth1 immunoblots indicated that Ftl, migrating at ∼25 kD, and Fth1, migrating at ∼22 kD, were more abundant in liver lysates and retentates from mice on the Fe-rich diet (Fig. S3*B*, bottom two images, lanes 1, 2, 7, and 8). Fe staining of native gels indicated that the Fe-stainable band was found only in the retentate of lysates from mice on the Fe-rich diet (Fig. S3*C*). (Although the pellet also contained immunoreactive Ftl1 and Fth1 (Fig. S3*B*, bottom two images, lanes 3 and 4), we could not determine if the pellet contained a stainable Fe band—pellets required solubilization with 8 M urea, which may have denatured ferritin.) Taken together, the precipitation/denaturation/filtration results strongly suggested that the Fe-stainable band represents ferritin.

**Figure 3:**
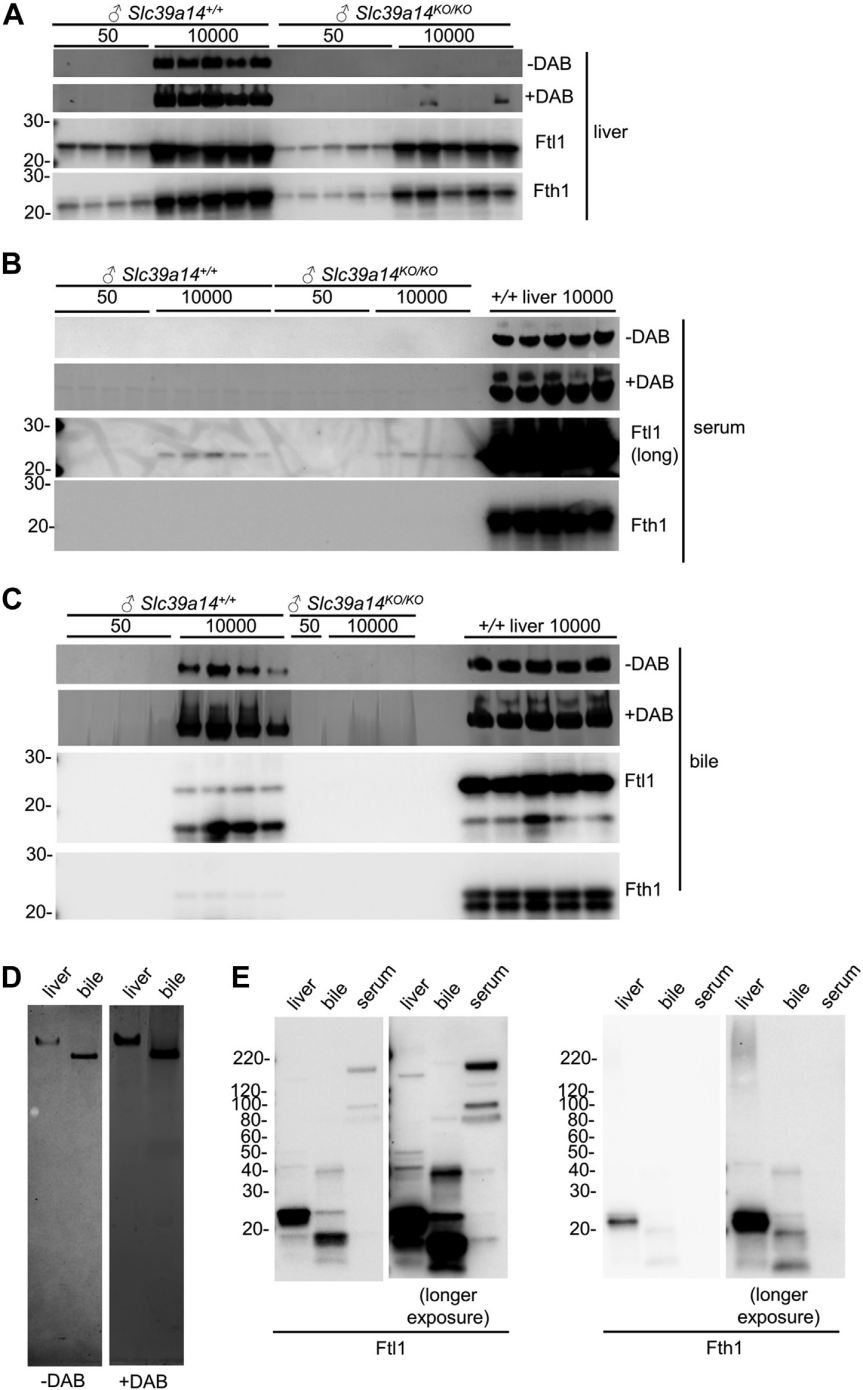
**Slc39a14 deficiency impairs biliary excretion of Fe-rich ferritin in mice on Fe-rich diet.***A*–*C*, in each panel, *top two images*: Fe staining of native gels of 100 μg liver lysates (*A*), 1 μl serum (*B*), and 10 μl bile (*C*) from male *Slc39a14* mice. In each panel, *bottom two images*: Ftl1 and Fth1 immunoblots of denaturing, reducing gels of 25 μg liver lysates (*A*), 1 μl serum (*B*) and 1 μl bile (*C*). Liver lysates from *Slc39a14*^*+/+*^ mice on Fe-rich diet included for reference in (*B* and *C*); 100 μg for Fe-stained gels and 25 μg for immunoblots. *D*, Fe staining of native gel of 25 μg liver lysate and 5 μl bile from a male *Slc39a14*^*+/+*^ mouse on Fe-rich diet. *E*, Ftl1 and Fth1 immunoblots of denaturing, reducing gels of 10 μg liver lysate, 1 μl bile, and 1 μl serum from a male *Slc39a14*^*+/+*^ mouse on Fe-rich diet. Longer exposures in *right panels*.

To determine if the bile was enriched in Fe-loaded ferritin, we next analyzed the liver, serum, and bile with Fe-stained native gels and Ftl1 and Fth1 immunoblots (Fig. 3, *A*–*C*, uncropped images in Fig. S4). (Immunoblots were also used to validate ELISA-based Ftl1 and Fth1 measurements reported above.) For liver lysates, Fe-stainable bands were prominent only in *Slc39a14*^*+/+*^ mice on Fe-rich diets (Fig. 3*A*, top two images). Ftl1 and Fth1 were most abundant in mice on Fe-rich diets with bands more intense in *Slc39a14*^*+/+*^ mice (Fig. 3*A*, bottom two images). For serum, Fe-stainable bands and Fth1 were not observed and faint Ftl1 bands were detected only in mice on Fe-rich diets (Fig. 3*B*). For bile, Fe-stainable bands and prominent Ftl1 bands were observed only in *Slc39a14*^*+/+*^ mice on Fe-rich diets (Fig. 3*C*). (Because of limited bile volumes, the bile blot was probed for Ftl1, then stripped and probed for Fth1. The additional bands observed in the Fth1 western in liver lysates may reflect residual binding of anti-Ftl1 antibodies.) These results suggested that bile ferritin is loaded with Fe.

In the above blots, we observed that the Fe-stainable band appeared to migrate faster in the bile than liver. We examined this by directly comparing the migration patterns of the Fe-stainable band and immunoreactive Ftl1 and Fth1 in the liver, bile, and serum. The Fe-stainable band from Fe-loaded *Slc39a14*^*+/+*^ bile migrated more quickly than that from the liver (Fig. 3*D*). The most prominent Ftl1 bands from Fe-loaded *Slc39a14*^*+/+*^ bile and sera migrated more quickly than those from the liver (Fig. 3*E*). These results suggested that bile ferritin is biochemically distinct from ferritin from other compartments. This is consistent with the observation made above that bile ferritin has a higher Ftl1:Fth1 ratio than liver or serum ferritin (Fig. 2*H*).

We also considered that methanol/heat treatment could enrich for ferritin in the bile and permit measurement of nonheme Fe levels in bile retentates as a quantitative assessment of bile ferritin Fe levels. However, methanol/heat treatment removed minimal protein content from the bile (Fig. S5, top two images, lanes 1–4 *versus* 9–12). As such, this approach was not appropriate for measurements of bile ferritin-bound Fe.

### Heme may contribute to biliary Fe in mice on Fe-rich diets

While ferritin was abundant in Fe-rich bile, we also assessed if heme Fe was also present in the bile. Given that bile volumes were very limited at this point in our study, we analyzed the pooled bile sample from male *Slc39a14*^*+/+*^ mice on the Fe-rich diet used in Fig. S5. Nonheme Fe levels were 32.3 μg/ml or 579.8 μM, while heme and hemin levels were 83.6 and 142.1 μM respectively. We also estimated the amount of bile heme Fe possibly derived from RBC lysis, perhaps introduced into bile during surgical collection. To do this, we measured Hba levels, although this calculation does assume that Hba in the bile derives from lysis of RBCs present in bile. Hba levels were 40.4 μg/ml bile. Assuming a molecular weight of 15 kD for Hba, this equates to 2.7 μM Hba (Table S1). If every 2 mol Hba corresponded to 1 mol hemoglobin bound to 4 mol Fe, the bile contained at maximum 5.4 μM RBC-derived heme Fe. Since heme and hemin levels were much greater than 5 μM, this approximation suggests that most heme Fe in bile is not derived from RBC lysis.

### Slc30a10 deficiency does not impair biliary Fe excretion in mice on Fe-rich diet

**Figure 4:**
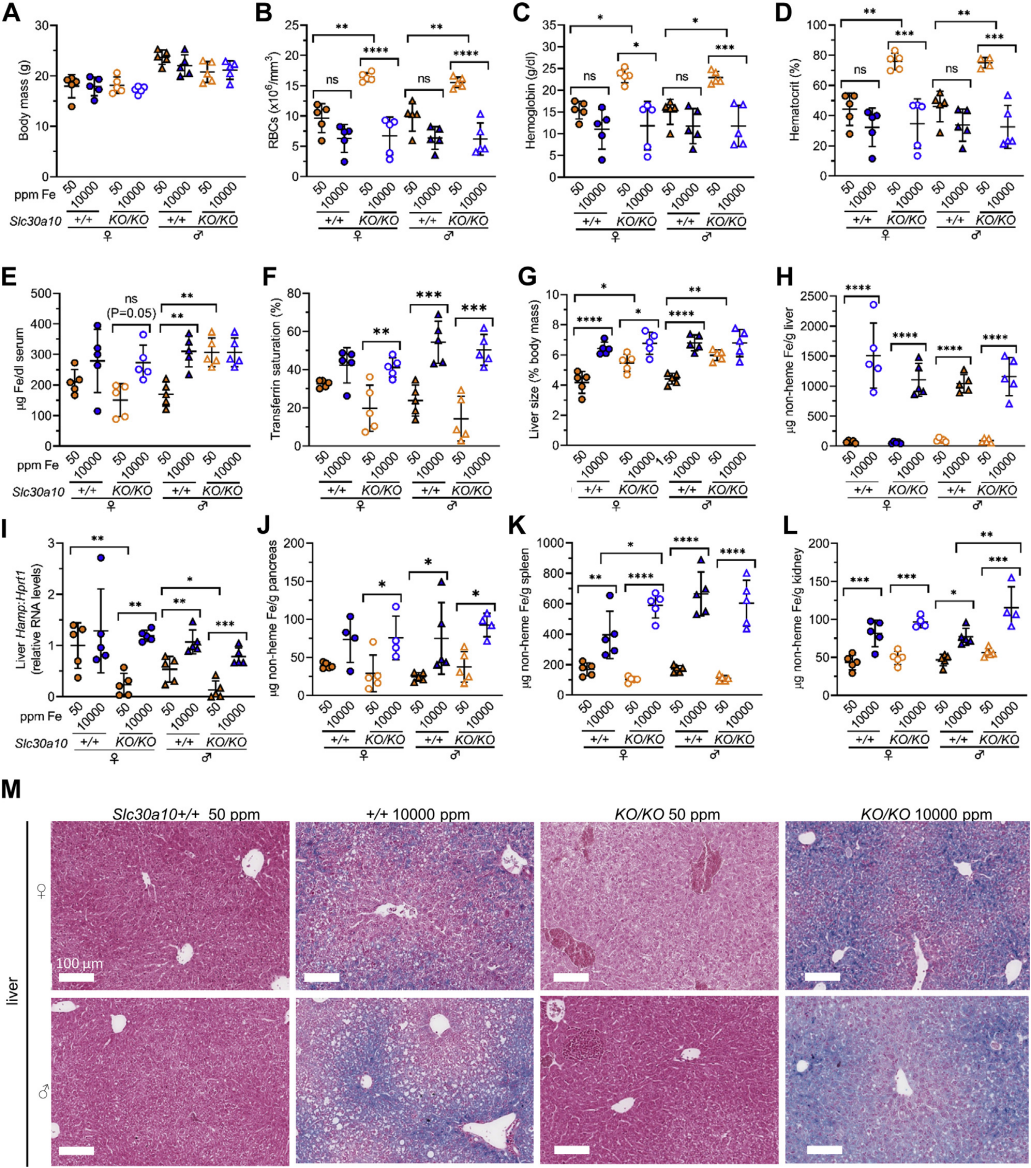
**Slc30a10 deficiency does not impair liver Fe loading.***Slc30a10*^*+/+*^ and *Slc30a10*^*KO/KO*^ mice were weaned onto Fe-sufficient or -rich diets, then underwent bile, blood, and tissue collection at 2 months old. *A*, body mass. *B*, red blood cell (RBC) counts. *C*, hemoglobin levels. *D*, hematocrits. *E*, serum Fe levels. *F*, transferrin saturations. *G*, liver mass as percent of body mass. *H*, liver non-heme Fe levels. *I*, liver hepcidin (*Hamp*) RNA levels relative to *Hprt1* RNA levels; values in each group normalized to average value in female *Slc39a14*^*+/+*^ mice on 50 ppm Fe diet. *J*–*L*, nonheme Fe levels in pancreas (*J*), spleen (*K*), and kidney (*L*). *M*, liver Fe stains at 10×; scale bar indicates 100 μm. Bars indicate mean ± standard deviation. Groups within each sex compared by one-way ANOVA with Tukey’s post-hoc test as in Figure 1.

While SLC39A14 imports Mn from the blood into hepatocytes, SLC30A10 exports Mn from hepatocytes into the bile. Given that SLC39A14 also imports excess Fe into hepatocytes, we considered that SLC30A10 may export excess Fe from hepatocytes into the bile. To test this, *Slc30a10*^*+/+*^ and *Slc30a10*^*KO/KO*^ mice were weaned onto diets containing 50 or 10,000 ppm Fe, then harvested for the bile, blood, and tissues at 2 months old. We first analyzed the mice for basic parameters of Fe homeostasis. The Fe-rich diet had no impact on body mass (Fig. 4*A*). We next assessed blood parameters. Unlike SLC39A14 deficiency, SLC30A10 deficiency causes erythropoietin excess and polycythemia, although the link between Mn excess and erythropoietin excess has yet to be firmly established. The Fe-rich diet decreased RBC counts, hemoglobin levels, and hematocrits in *Slc30a10*^*+/+*^ mice, but these decreases were not significant (Fig. 4, *B*–*D*). The Fe-rich diet decreased RBC counts, hemoglobin levels, and hematocrits in *Slc30a10*^*KO/KO*^ mice to the same levels seen in *Slc30a10*^*+/+*^ mice on the Fe-rich diet (Fig. 4, *B*–*D*). (The significance of this will be discussed below.) The Fe-rich diet also increased serum Fe levels in male *Slc30a10*^*+/+*^ mice and transferrin saturations in all except female *Slc30a10*^*+/+*^ mice (Fig. 4, *E* and *F*).

We next analyzed tissue Fe levels. The Fe-rich diet increased liver masses in all mice except male *Slc30a10*^*KO/KO*^ mice (Fig. 4*G*), similar to what was observed for *Slc39a14* mice. The Fe-rich diet increased liver nonheme Fe and hepcidin RNA levels in all mice except for hepcidin levels in female *Slc30a10*^*+/+*^ mice (Fig. 4, *H* and *I*). Notably, liver hepcidin RNA levels were lower in *Slc30a10*^*KO/KO*^ than *Slc30a10*^*+/+*^ mice on Fe-sufficient diets, the implications of which will be discussed below. The Fe-rich diet increased nonheme Fe levels in the pancreas, spleen, and kidney in all mice except for the pancreas in female *Slc30a10*^*+/+*^ mice (Fig. 4, *J*–*L*). Heart nonheme Fe levels were not impacted by diet (data not shown). With regard to tissue Fe staining, Fe was most abundant in periportal hepatocytes in all mice on Fe-rich diets (Fig. 4*M*), in contrast to the staining patterns observed for *Slc39a14* mice. Fe staining was not observed in pancreatic cells but was detected in splenic red pulp macrophages in mice on Fe-rich diets (Fig. S6). Faint Fe staining was observed near the apical surface of enterocytes or within the central axis of the villi in some of the mice on Fe-rich diets, although intensity varied regionally even within samples.

**Figure 5:**
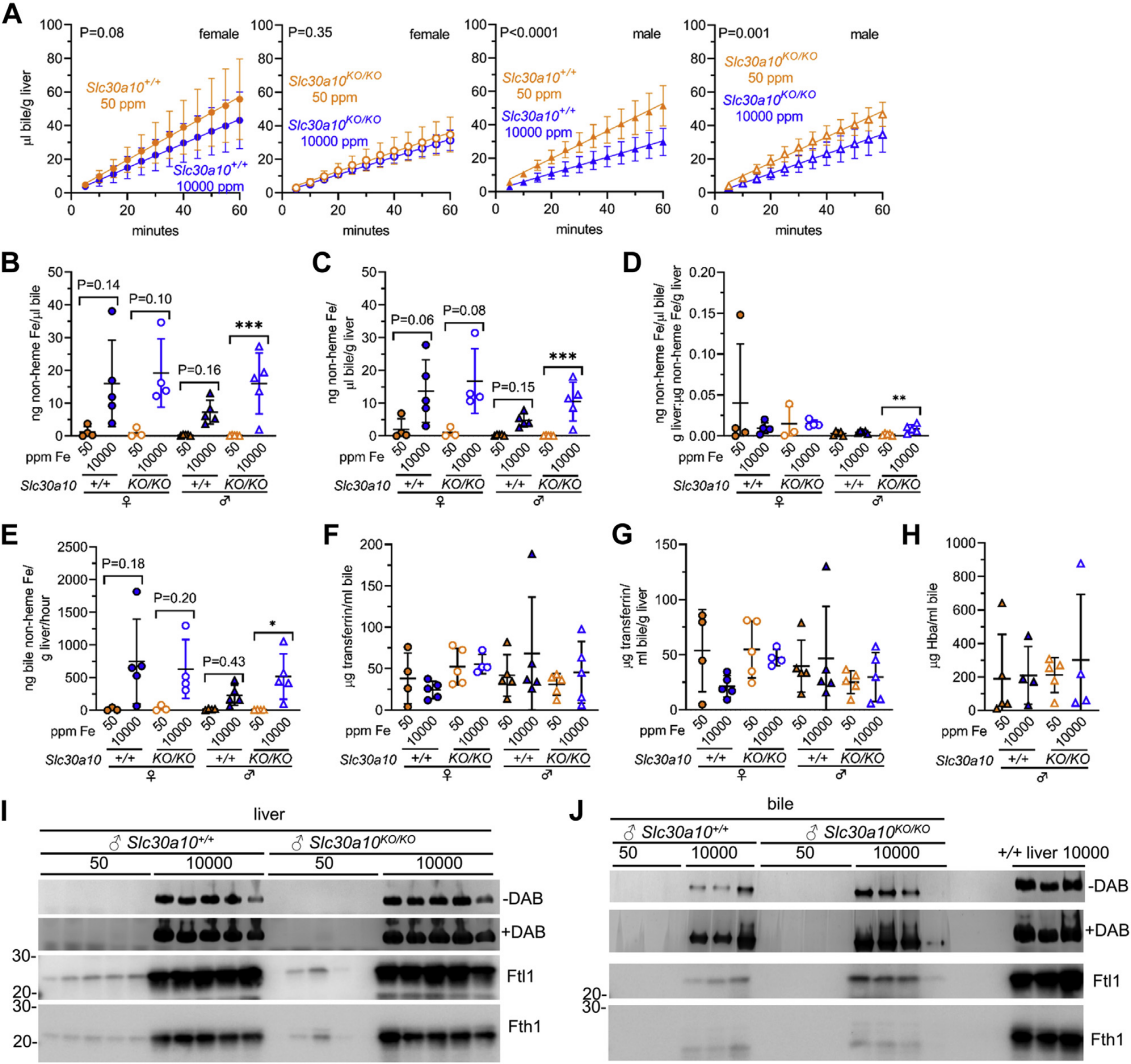
**Slc30a10 deficiency does not impair liver Fe loading or biliary excretion of excess Fe.***Slc30a10*^*+/+*^ and *Slc30a10*^*KO/KO*^ mice were weaned onto Fe-sufficient or -rich diets, then underwent bile, blood, and tissue collection at 2 months old. *A*, bile flow rates, curves compared by linear regression. *B* and *C*, bile nonheme Fe levels without (*B*) and with (*C*) normalization to liver mass. *D*, ratios of bile to liver nonheme Fe levels. *E*, bile nonheme Fe levels normalized to liver mass, excreted per hour. *F* and *G*, bile transferrin levels without (*F*) and with (*G*) normalization to liver mass. *H*, bile Hba levels. *I* and *J*, Fe stained native gels and Ftl1 and Fth1 denaturing immunoblots of liver lysates (*I*) and bile (*J*), presented as in Figure 3, *A* and *C*. Bars indicate mean ± standard deviation. Groups within each sex compared by one-way ANOVA with Tukey’s post-hoc test as in Figure 1.

Given that the Fe-rich diet increased tissue Fe levels, we next analyzed bile collected surgically from mice at the time of harvests. The Fe-rich diet decreased bile flow rates in male but not female *Slc30a10* mice (Fig. 5*A*). Bile flow rates were also lower in female *Slc30a10*^*KO/KO*^ than *Slc30a10*^*+/+*^ mice on either diet (*p* values not shown in Fig. 5*A*). The Fe-rich diet increased bile nonheme Fe levels with and without normalization to liver mass, but this did not reach significance in most mice (Fig. 5, *B* and *C*). (There were inadequate volumes of bile from female mice for all analyses, which limited the power to detect significant differences here.) The Fe-rich diet did not impact ratios of bile to liver nonheme Fe levels for most groups (Fig. 5*D*). The Fe-rich diet increased the amount of bile Fe excreted per hour, but the increases were not significant for most groups (Fig. 5*E*). Bile Tf levels were ∼25 to 50 μg protein/ml bile (and ∼25–50 μg protein/ml bile/g liver) in all groups (Fig. 5, *F* and *G*). Bile Hba levels did not differ between groups (Fig. 5*H*), even though *Slc30a10*^*KO/KO*^ mice had increased RBC counts. Prominent Fe-stainable, Ftl1, and Fth1 bands were detected in liver lysates and bile from *Slc30a10*^*+/+*^ and *Slc30a10*^*KO/KO*^ mice on Fe-rich diets, although band intensity varied between bile samples (Fig. 5, *I* and *J*; uncropped images in Fig. S7). Overall, our analyses indicated that Slc30a10 deficiency had no impact on tissue or bile Fe loading in mice on Fe-rich diets.

### Mn excess is attenuated in Slc39a14^KO/KO^ and Slc30a10^KO/KO^ mice on Fe-rich diet

**Figure 6:**
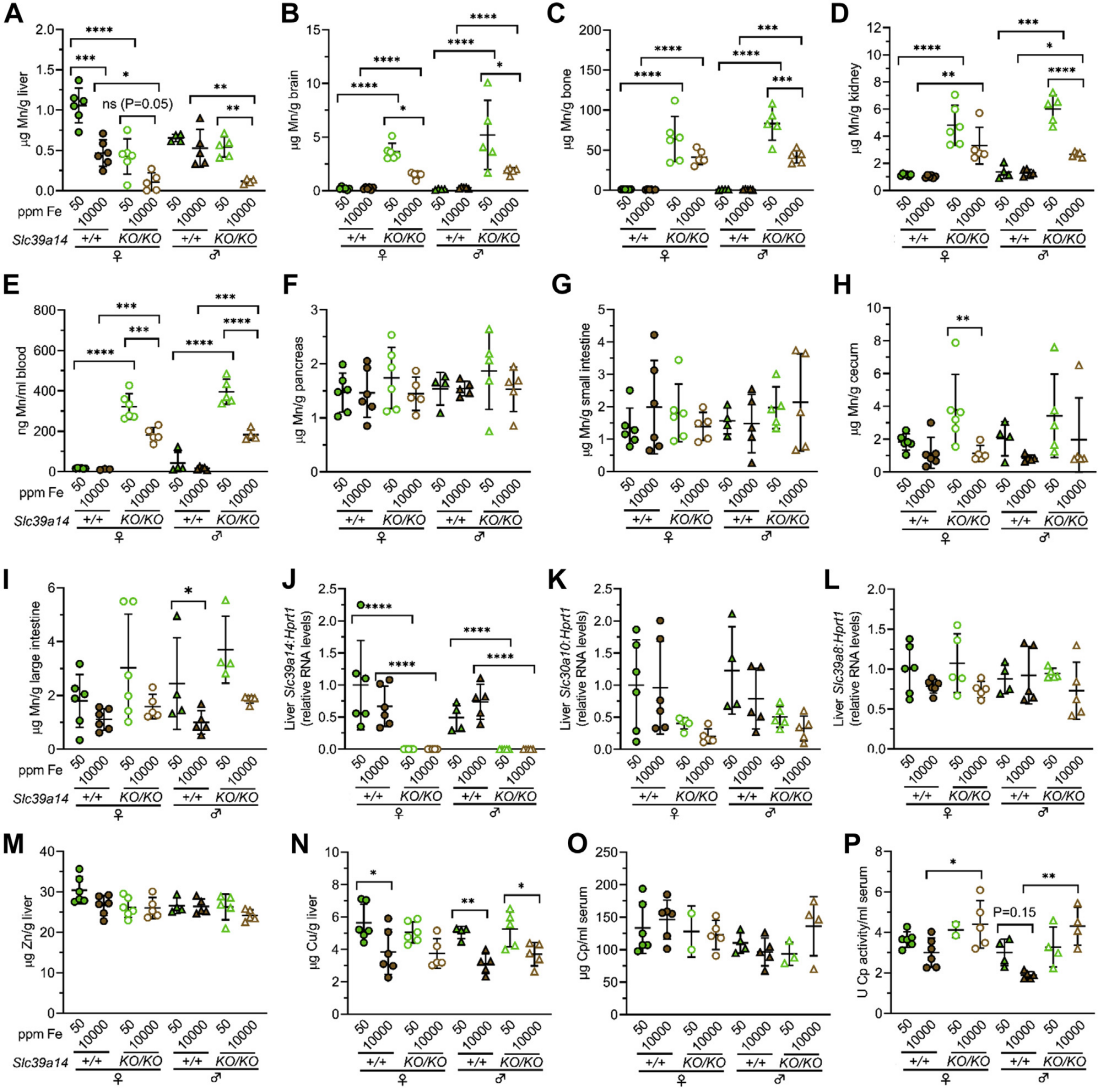
**Mn excess is attenuated in *Slc39a14***^***KO/KO***^**mice raised on an Fe-rich diet.***Slc39a14*^*+/+*^ and *Slc39a14*^*KO/KO*^ mice were weaned onto Fe-sufficient or -rich diets, then underwent collection of bile, blood, and tissues at 2 months of age. *A*–*I*, total Mn levels in liver (*A*), brain (*B*), bone (*C*), kidney (*D*), blood (*E*), pancreas (*F*), small intestine (*G*), cecum (*H*), and large intestine (*I*). *J*–*L*, liver *Slc39a14* (*J*), *Slc30a10* (*K*), and *Slc39a8* (*L*) RNA levels relative to *Hprt1* RNA levels, with values in each group normalized to average value in female *Slc39a14*^*+/+*^ mice on Fe-sufficient diet. *M* and *N*, zinc (Zn) (*M*) and copper (Cu) (*N*) levels in liver. *O* and *P*, serum ceruloplasmin (Cp) protein (*O*) and activity (*P*) levels. Bars indicate mean ± standard deviation. Groups within each sex were compared by one-way ANOVA with Tukey’s post-hoc test as in Figure 1.

In this study, we employed *Slc39a14*^*KO/KO*^ mice to analyze biliary excretion of excess Fe because SLC39A14 imports Fe into hepatocytes and to our knowledge it is the only Fe transporter shown to be specific to NTBI *in vivo*. However, under physiologic conditions, SLC39A14 transports other metals, including Mn and Zn. SLC39A14 deficiency results in systemic Mn excess although the liver, pancreas, and intestines are spared due to the essential role of SLC39A14 in Mn import into these organs. Notably, oral Fe has been used to treat patients with SLC39A14 deficiency (28, 29), as it is believed to outcompete Mn for absorption from the gut. Given this, we assessed if the Fe-rich diets altered Mn levels in *Slc39a14*^*KO/KO*^ mice. Liver Mn levels were lower in *Slc39a14*^*KO/KO*^ than *Slc39a14*^*+/+*^ mice on either diet except for male mice on the Fe-sufficient diet (Fig. 6*A*). The Fe-rich diet decreased liver Mn levels in all mice except for male *Slc39a14*^*+/+*^ mice, although the decrease in female *Slc39a14*^*KO/KO*^ mice did not reach significance. The Fe-rich diet had no impact on Mn levels in the brain, bone, kidney, and blood in *Slc39a14*^*+/+*^ mice but did attenuate Mn excess in the brain, bone, kidney, and blood Mn excess in *Slc39a14*^*KO/KO*^ mice except for the bone and kidney in female *Slc39a14*^*KO/KO*^ mice (Fig. 6, *B*–*E*). Mn levels in the pancreas, small intestine, cecum, and large intestine were not impacted for most groups (Fig. 6, *F*–*I*). To determine if the Fe-rich diet affected expression of Mn transport proteins, we measured liver RNA levels of *Slc39a14*, *Slc30a10*, and *Slc39a8*. (SLC39A8 imports Mn from bile into hepatocytes. SLC39A8 deficiency results in a rare inherited disease of Mn deficiency.) While *Slc39a14* RNA levels were lower in *Slc39a14*^*KO/KO*^ mice as expected, the Fe-rich diet otherwise had no impact on liver RNA levels of *Slc39a14*, *Slc30a10*, or *Slc39a8* (Fig. 6, *J*–*L*).

We also analyzed liver zinc and copper levels. The Fe-rich diet had no impact on zinc levels but did decrease copper levels in all mice except for female *Slc39a14*^*KO/KO*^ mice (Fig. 6, *M* and *N*). To determine if altered copper levels were functionally relevant, we measured serum levels and activity of ceruloplasmin, a copper-dependent ferroxidase abundantly expressed by the liver. The Fe-rich diet had no impact on serum ceruloplasmin levels or activity.

**Figure 7:**
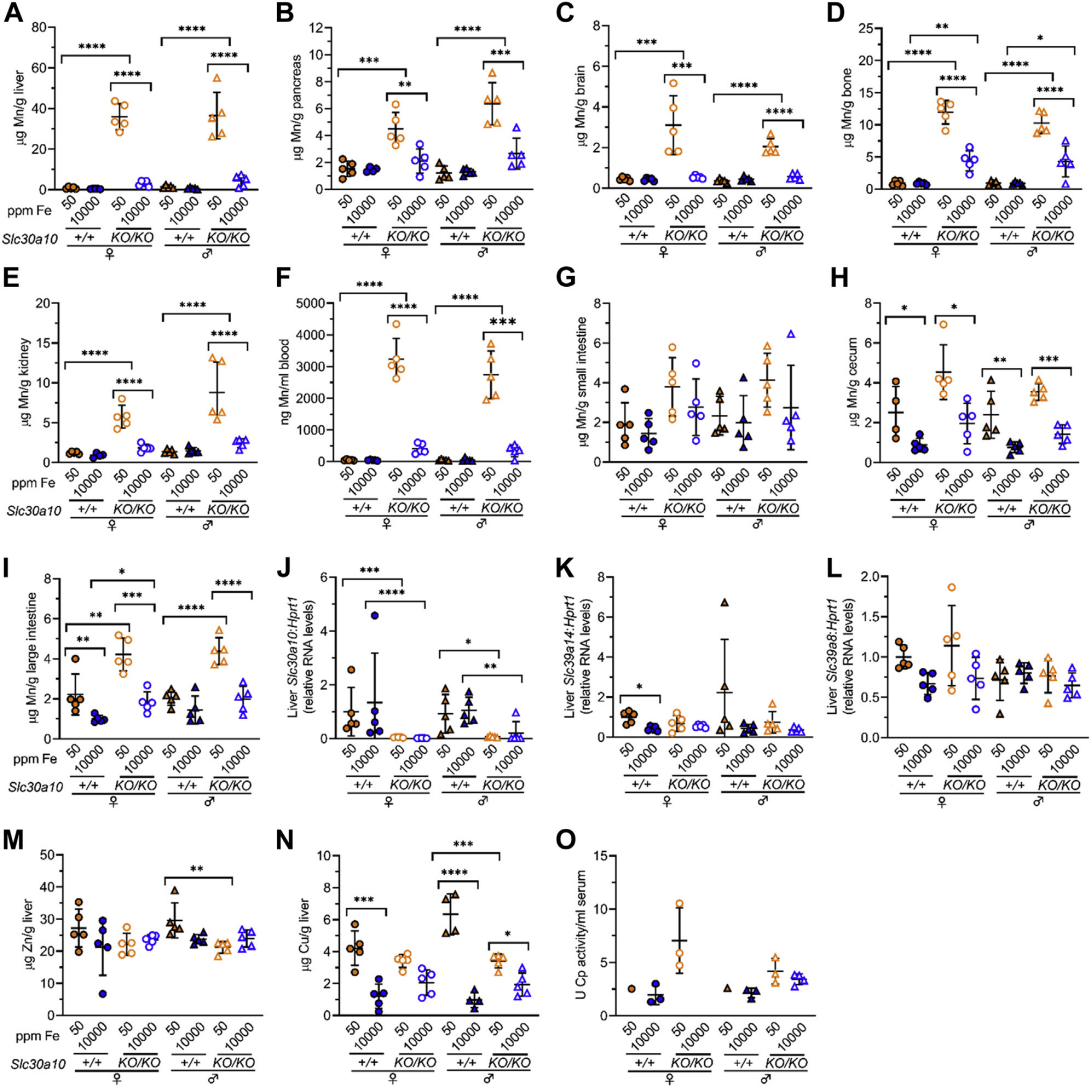
**Mn excess is attenuated in *Slc30a10***^***KO/KO***^**mice raised on an Fe-rich diet.***Slc30a10*^*+/+*^ and *Slc30a10*^*KO/KO*^ mice were weaned onto Fe-sufficient or -rich diets, then underwent collection of bile, blood, and tissues at 2 months of age. *A*–*I*, total Mn levels in liver (*A*), pancreas (*B*), brain (*C*), bone (*D*), kidney (*E*), blood (*F*), small intestine (*G*), cecum (*H*), and large intestine (*I*). *J*–*L*, liver *Slc30a10* (*J*), *Slc39a14* (*I*), and *Slc39a8* (*L*) RNA levels relative to *Hprt1* RNA levels, with values in each group normalized to average value in female *Slc39a14*^*+/+*^ mice raised on 50 ppm Fe diet. *M* and *N*, zinc (Zn) (*M*) and copper (Cu) (*N*) levels in liver. *O*, serum ceruloplasmin (Cp) activity levels. Bars indicate mean ± standard deviation. Groups within each sex were compared by one-way ANOVA with Tukey’s post-hoc test as in Figure 1.

Like SLC39A14 deficiency, SLC30A10 deficiency also results in Mn excess, and oral Fe supplementation attenuates disease characteristics in patients (28, 29). To determine if the Fe-rich diet attenuated Mn excess in *Slc30a10*^*KO/KO*^ mice, we measured Mn levels in *Slc30a10*^*KO/KO*^ mice. (As shown above, the Fe-rich diet normalized aberrant RBC parameters in *Slc30a10*^*KO/KO*^ mice.) While the Fe-rich diet had no impact on Mn levels in the liver, pancreas, brain, bone, kidney, and blood in *Slc30a10*^*+/+*^ mice, it did attenuate Mn excess in these compartments in *Slc30a10*^*KO/KO*^ mice (Fig. 7, *A*–*F*). Mn levels in small intestines were not impacted by genotype or diet (Fig. 7*G*). The Fe-rich diet also decreased Mn levels in the cecum and large intestine of all mice except for the large intestine in male *Slc30a10*^*+/+*^ mice (Fig. 7, *H* and *I*). As performed above, we also measured liver RNA levels of *Slc39a14*, *Slc30a10*, and *Slc39a8*. *Slc30a10* RNA levels were lower in *Slc30a10*^*KO/KO*^ than *Slc30a10*^*+/+*^ mice on either diet as expected (Fig. 7*J*). No differences were noted in other RNA levels except for decreased *Slc39a14* RNA levels in female *Slc30a10*^*+/+*^ mice raised on the Fe-rich diet (Fig. 7, *K* and *L*).

We also analyzed liver zinc and copper levels. The Fe-rich diet had no impact on zinc levels but did decrease copper levels in all mice except for female *Slc30a10*^*KO/KO*^ mice (Fig. 7, *M* and *N*). Ceruloplasmin activity was not impacted by the Fe-rich diet, although we had limited sample volumes for this analysis (Fig. 7*O*).

## Discussion

To establish the molecular basis of biliary Fe excretion, we exploited the finding first reported by Jenkitkasemwong *et al.* (30) that Slc39a14 is essential for development of liver Fe overload in mouse models of Fe excess. To determine if Slc39a14-dependent Fe import into hepatocytes is a prerequisite for biliary excretion of excess Fe, we raised *Slc39a14*^*+/+*^ and *Slc39a14*^*KO/KO*^ mice on Fe-sufficient and -rich diets.

We first analyzed basic parameters of Fe homeostasis. Our analysis of tissue Fe levels in *Slc39a14* mice produced results similar to those previously reported (30). In mice raised on the Fe-rich diet, Slc39a14 deficiency attenuated liver Fe excess and increased Fe levels in extrahepatic organs. Liver Fe was most abundant in periportal hepatocytes in *Slc39a14*^*+/+*^ mice and nonparenchymal cells in *Slc39a14*^*KO/KO*^ mice. This is consistent with the essential role of Slc39a14 in import of excess Fe into hepatocytes. In this study, we also considered if Slc30a10 is essential for export of excess Fe into the bile by raising *Slc30a10*^*+/+*^ and *Slc30a10*^*KO/KO*^ mice on Fe-sufficient and -rich diets. Analysis of basic phenotypes of Fe homeostasis showed that the Fe-rich diet led to hepatic and extrahepatic Fe excess with liver Fe most abundant in periportal hepatocytes in all *Slc30a10* mice. In contrast to Slc39a14 deficiency, Slc30a10 deficiency did not attenuate the development of liver Fe excess in mice on Fe-rich diets. This was expected, given that Slc30a10 is not known to play a role in liver Fe import.

With basic parameters of Fe homeostasis established in *Slc39a14* and *Slc30a10* mice raised on Fe-sufficient and -rich diets, we next proceeded to analyze the bile. This produced several observations. The Fe-rich diet decreased bile flow rates in most mice, which may reflect Fe toxicity. Bile nonheme Fe levels increased in all Fe-loaded mice except for *Slc39a14*^*KO/KO*^ mice, indicating that Slc39a14, but not Slc30a10, is essential for biliary excretion of excess Fe in a setting of dietary Fe loading. The Fe-rich diet did not result in hepatocyte Fe loading or increased bile Fe levels in *Slc39a14*^*KO/KO*^ mice, suggesting that bile nonheme Fe is derived from hepatocytes in mice raised on an Fe-rich diet. Tf levels could only account for a small fraction of bile nonheme Fe in Fe-loaded wild-type mice. Fe-loaded ferritin, comprised largely of Ftl1, was enriched in the bile from Fe-loaded wild-type (and *Slc30a10*^*KO/KO*^) mice at levels sufficient to account for all nonheme Fe. While our study did not address the mechanism of Fe export into bile, Fe-loaded ferritin is most likely exported into the bile *via* lysosomal exocytosis as previously shown (16, 17, 18, 19, 20, 21). Biliary excretion of excess Fe is unlikely to involve nuclear receptor coactivator 4 (NCOA4), as NCOA4 delivers ferritin to lysosomes in Fe deficiency, not excess (40). Ferroportin, the only known mammalian Fe exporter, is also unlikely to export Fe into the bile, given that it localizes to the hepatocyte sinusoidal membrane (41).

We also detected heme and hemin, an oxidized form of heme, in Fe-rich bile. Our estimates suggested that only a small fraction of bile heme Fe could originate from RBC lysis. One key question is the route by which heme enters the bile. If not by lysosomal exocytosis, heme may enter the bile by transport. Feline leukemia virus subgroup C cellular receptor 1a (FLVCR1a) is a heme exporter but is unlikely to contribute. It localizes to the sinusoidal membrane when overexpressed in HepG2 cells, and hepatic Flvcr1a deficiency does not impair export of injected heme into bile (42). BCRP/ABCG2, a protoporphyrin transporter localizing to the hepatocyte canalicular membrane, is a more likely candidate. While one group reported that bile protoporphyrin IX levels do not differ between wild-type and Abcg2-deficient mice, another demonstrated the Abcg2-deficient mice have impaired export of injected protoporphyrin IX into the bile (43, 44).

While analyzing bile ferritin, we also analyzed serum ferritin for comparison. Serum Ftl1 and Fth1 levels were lower in *Slc39a14*^*KO/KO*^ than *Slc39a14*^*+/+*^ mice on Fe-rich diets. Given that hepatocytes did not load with Fe in *Slc39a14*^*KO/KO*^ mice raised on the Fe-rich diet, the decrease in serum ferritin levels in *Slc39a14*^*KO/KO*^ mice on Fe-rich diets suggests that most serum ferritin is produced by hepatocytes in conditions of dietary Fe loading.

In our study, we also considered the effect of Fe-rich diets on Mn. As mentioned above, both *Slc39a14*^*KO/KO*^ and *Slc30a10*^*KO/KO*^ mice are models of inherited diseases of Mn excess. The Fe-rich diet attenuated Mn excess in *Slc39a14*^*KO/KO*^ and *Slc30a10*^*KO/KO*^ mice. This result is similar to the observation that oral Fe attenuates severity of disease in patients with SLC39A14 or SLC30A10 deficiency, although the Fe levels used in our study were much greater than therapeutic doses. The Fe-rich diet also normalized RBC counts, hemoglobin levels, and hematocrits levels in *Slc30a10*^*KO/KO*^ mice to levels seen in *Slc30a10*^*+/+*^ mice on the same diet. In contrast to SLC39A14 deficiency, SLC30A10 deficiency results in polycythemia and excess levels of erythropoietin, a hormone that stimulates RBC synthesis. Erythropoietin excess is attributed to a stimulatory effect of Mn excess on erythropoietin expression (28, 29). The observation that the Fe-rich diet attenuated both Mn excess and aberrant RBC parameters is consistent with the notion that Mn excess drives increased erythropoiesis in SLC30A10 deficiency.

Another novel observation in our study is decreased liver hepcidin expression in *Slc30a10*^*KO/KO*^ mice. Hepcidin levels increased in *Slc30a10*^*KO/KO*^ mice on Fe-rich diets suggesting that hepcidin deficiency in untreated mice reflects aberrant Fe and/or Mn levels. Another possible contributor is erythropoietin excess, which suppresses hepcidin expression. The possible link between Mn excess, erythropoietin excess, and hepcidin deficiency in SLC30A10 deficiency requires further investigation.

**Figure 8:**
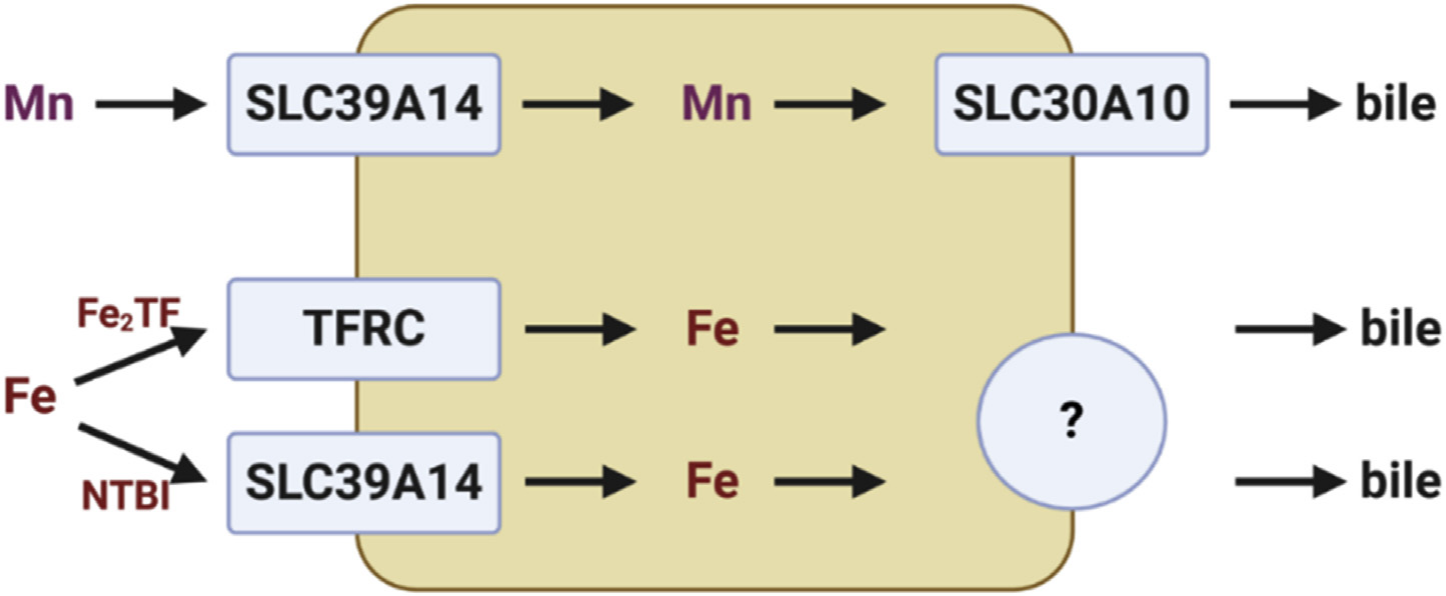
**Model.** Under physiologic conditions, Mn is imported into hepatocytes by SLC39A14, then exported into bile by SLC30A10, while diferric transferrin (Fe_2_TF) is imported into hepatocytes by transferrin receptor (TFRC)-mediated endocytosis. Under conditions of Fe excess, non-transferrin-bound Fe (NTBI) is imported into hepatocytes by SLC39A14; excess iron may be exported into bile *via* lysosomal exocytosis. Image created with Biorender.

Overall, our study demonstrates that import of excess Fe into hepatocytes by SLC39A14 is essential for biliary Fe excretion in conditions of Fe excess induced by dietary means (Fig. 8). Excess Fe is transported into the bile as ferritin and heme. Future studies will interrogate the role of biliary Fe excretion in mouse models of inherited Fe excess, as dietary Fe loading is not a common cause of Fe excess. Future studies will also interrogate lysosomal exocytosis and other mechanisms of biliary Fe export, particularly in the setting of inherited diseases of Fe excess. Fe excess in hereditary hemochromatosis and other inherited diseases is caused by hepcidin deficiency and excessive dietary Fe absorption. The observation that increased absorption leads to systemic Fe excess indicates that biliary excretion of excess Fe is insufficient to counter the increased Fe absorption observed under conditions of hepcidin deficiency. However, NTBI undergoes enterohepatic circulation. The molecular mechanism of enterohepatic circulation of Fe is not known. If hepcidin deficiency increases rates of enterohepatic circulation of Fe, hepcidin deficiency would limit the impact of biliary Fe excretion on elimination of excess Fe from the body. This notion has yet to be tested experimentally. Finally, we propose that biliary excretion and enterohepatic circulation of excess Fe may have clinical significance. Even though increased dietary Fe absorption is a key determinant of Fe excess in conditions of hepcidin deficiency, both biliary excretion and enterohepatic circulation may be amenable to pharmacologic targeting to increase rates of biliary Fe elimination as a potential novel treatment for diseases of Fe excess.

## Experimental procedures

### Care and generation of mice and sample collection

Mouse work was approved by the Institutional Animal Care and Use Committee at Brown University. *Slc39a14*^*+/KO*^ mice were generated on a 129+Ter/SvJcl x C57BL/6 background (45). *Slc30a10*^*+/KO*^ mice were generated on a C57BL/6N background (36). Mice were bred and maintained in the animal facility at Brown University. Mice were group-housed in ventilated cage racks, maintained on a 12-h light/12-dark cycle with controlled temperature and humidity, and provided standard chow (LabDiet 5010; 120 ppm Mn, 250 ppm Fe) and water *ad libitum*. Heterozygous mice were bred to generate wild-type and mutant homozygous mice. Littermates of same sex were randomly assigned to experimental groups. At weaning, mice were fed AIN-93G diet containing 50 ppm Fe (Envigo, TD.130018) or 10,000 ppm Fe (Envigo, TD.130015) as previously performed (30). At 2-months-old, mice underwent bile, blood, and tissue collection as previously described, with bile collected by ligation of the common bile duct, cannulation of the gallbladder, and collection over 60 min (36). Blood was collected by retro-orbital puncture into EDTA-coated tubes (BD) using heparinized capillary tubes (Fisher) then into serum collection tubes (BD) using nonheparinized capillary tubes (Fisher). Mice were euthanized by cervical dislocation and tissues collected for histology and metal, DNA, RNA, and protein analysis. (Intestines were washed of lumenal contents, and intestine fragments were used for metal analysis.) Tissues for histology were fixed in 10% phosphate-buffered formalin (Fisher) overnight and stored in 70% ethanol at 4 °C.

### Sample analysis (except mass spectrometry and gel electrophoresis)

Tissue nonheme Fe levels were measured by digesting 10 to 200 mg tissue in 1 ml 3 N hydrochloric acid (Fisher)/10% trichloroacetic acid (Millipore Sigma) at 65 °C for 2 days, with 30 min vortexing each day, followed by centrifugation. Fe levels were measured by mixing 10 μl supernatants with 200 μl chromagen (five volumes MilliQ water; five volumes saturated sodium acetate (Fisher); one volume chromagen stock, consisting of 0.1% bathophenanthroline sulfonate (Millipore Sigma) and 1% thioglycolic acid (Millipore Sigma)) in a 96-well plate. Fe standards (Fisher) were included. After a 10-min incubation, absorbances were measured at 535 nm. Mock digests without samples were included for this and all other metal analyses. Bile nonheme Fe levels were measured by digesting 10 μl bile with 10 μl 20% trichloroacetic acid/6 N hydrochloric acid at 65 °C for 2 h, followed by centrifugation and spectrophotometric measurement as above.

Tissue Mn, zinc, and copper levels were measured by digestion of 10 to 200 mg tissue in 1 ml 70% trace metal grade nitric acid at 65 °C for 2 h, 25-fold dilution with MilliQ water, and analysis by inductively coupled plasma–atomic emission spectroscopy (ICP-AES) (Thermo Fisher Scientific, iCAP 7400 DUO) as previously described (36). Blood Mn levels were measured by digestion with two volumes 70% trace metal grade nitric acid (Fisher) at 65 °C for 2 h, 25-fold dilution with MilliQ water (Millipore Sigma), and analysis by graphite furnace atomic absorbance spectroscopy (GFAAS) (PerkinElmer, AAnalyst 600) as previously described (36).

Serum Fe levels and transferrin saturations were measured using Iron Colorimetric Assay Kit and UIBC Colorimetric Assay Kit (Adipogen). Complete blood counts were done on freshly collected anticoagulated blood using Vet Abc Plus (Scil). For histology, fixed tissues were embedded, sectioned, stained with Iron Stain Kit (Millipore Sigma), and scanned using Aperio ScanScope (Leica Biosystems).

ELISAs were performed using Mouse Transferrin ELISA Kit (Alpha Diagnostics International), Mouse Ferritin ELISA Kit (Abcam), Mouse Ferritin Heavy Chain ELISA Kit (Novus), and Mouse Hemoglobin ELISA Kit (Abcam). Heme and hemin levels were analyzed using Heme Assay Kits and Hemin Assay Kits (Millipore Sigma). Serum ceruloplasmin protein and activity levels were measured using Mouse Ceruloplasmin ELISA Kit (Novus) and Ceruloplasmin Colorimetric Activity Kit (Thermo Fisher).

For RNA analysis, 100 to 200 mg tissue was homogenized in TRIzol (ThermoFisher) using 0.5 mm zirconium beads and Bullet Blender (Next Advance), followed by chloroform extraction, isopropanol precipitation, and 70% ethanol wash, as previously described (36). Standards were made by serially diluting mixtures of control and experimental samples, then processed identically as experimental samples. Samples underwent DNase treatment and cDNA synthesis using the High Capacity cDNA Reverse Transcription Kit with RNase Inhibitor (ThermoFisher). qPCR was performed using PowerUP SYBR Green Master Mix (ThermoFisher) and primer pairs previously reported (36).

### Mass spectrometry

Bile samples (at least 25 μg protein/sample, with five samples from female *Slc39a14*^*+/+*^ mice on 50 ppm diets and five samples from female *Slc39a14*^*+/+*^ mice on 10,000 ppm diets) were digested on 10 kDa filters (Pall Corporation) using FASP digest procedure (46). After reduction and alkylation, samples were redissolved on top of filters in 100 μl 50 mM tri-ethyl ammonia bicarbonate buffer. After addition of 1 μg trypsin (Promega) to each sample and overnight digestion, peptide samples were spun and labeled with TMT11plex (Thermo Fisher) according to manufacturer’s protocol. Labeled samples were fractionated on Hi-pH columns (Thermo) to ten fractions according to vendor instructions. After separation, each fraction was submitted for single LC-MS/MS on a Lumos Tribrid (Thermo) equipped with 3000 Ultima Dual nanoHPLC pump (Thermo). Peptides were separated onto a 150 μm inner diameter microcapillary trapping column packed first with approximately 3 cm of C18 Reprosil resin (5 μm, 100 Å, Dr Maisch GmbH) followed by a 50 cm PharmaFluidics (Belgium) micropack analytical column. Separation was achieved by applying a gradient of 5 to 27% acetonitrile in 0.1% formic acid over 90 min at 200 nl/min. Electrospray ionization was enabled by applying a voltage of 1.8 kV using a home-made electrode junction at the end of the microcapillary column and sprayed from stainless-steel tips (PepSep). The Lumos Orbitrap was operated in data-dependent mode for the mass spectrometry methods. The mass spectrometry survey scan was performed in the Orbitrap in the range of 400 to 1800 m/z at a resolution of 6 × 10^4^, followed by the selection of the 20 most intense ions (TOP20) for CID-MS2 fragmentation in the ion trap using a precursor isolation width window of 2 m/z, AGC setting of 10,000, and a maximum ion accumulation of 50 ms. Singly charged ion species were not subjected to CID fragmentation. Normalized collision energy was set to 35 V and an activation time of 10 ms. Ions in a 10 ppm m/z window around ions selected for MS2 were excluded from further selection for fragmentation for 90 s. The same TOP20 ions were subjected to HCD MS2 event in the Orbitrap part of the instrument. The fragment ion isolation width was set to 0.8 m/z, AGC was set to 50,000, the maximum ion time was 150 ms, normalized collision energy was set to 34 V, and an activation time of 1 ms was set for each HCD MS2 scan. Raw data were submitted for analysis in Proteome Discoverer 2.4 (Thermo Scientific) software. Assignment of MS/MS spectra was performed using the Sequest HT algorithm by searching the data against a protein sequence database including all entries from the Human Uniprot database (SwissProt 19,768 2019) and other known contaminants such as human keratins and common lab contaminants. Sequest HT searches were performed using a 10 ppm precursor ion tolerance and requiring each peptide’s N-/C terminus to adhere with trypsin protease specificity, while allowing up to two missed cleavages. 11-plex TMT tags on peptide N termini and lysine residues (+229.163 Da) were set as static modifications while methionine oxidation (+15.99492 Da) was set as variable modification. A MS2 spectra assignment false discovery rate (FDR) of 1% on protein level was achieved by applying the target-decoy database search. Filtering was performed using a Percolator (64 bit version) (47). For quantification, a 0.02 m/z window centered on the theoretical m/z value of each the six reporter ions and the intensity of the signal closest to the theoretical m/z value was recorded. Reporter ion intensities were exported in the result file of Proteome Discoverer 2.4 search engine as an Excel table. The total signal intensity across all peptides quantified was summed for each TMT channel, and all intensity values were normalized to account for potentially uneven TMT labeling and/or sample handling variance for each labeled channel. (An Excel file containing normalized data from all TMT channels is included as Supplementary information). Statistical analysis was performed by an in-house R package-based program that uses code from Bioconductor for TMT mass spectrometry analysis.

### Protein electrophoresis

For gel analysis, 50 to 200 mg liver was homogenized in 50 mM Tris pH 7.4/150 mM NaCl/1% Triton X-100/5 mM EDTA/Halt protease inhibitors (Thermo), then centrifuged. Supernatants were assayed for protein using DC Protein Assay (BioRad). For Fe-stained gels, samples were electrophoresed on Criterion 4 to 20% TGX Stain-Free Gels (BioRad) under native conditions and imaged for protein using a ChemiDoc (BioRad). Gels were washed with MilliQ water for 5 min four times, stained for 10 min using Iron Stain Kit, imaged using a ChemiDoc, washed, stained using Sigmafast DAB with Metal Enhancer (Millipore Sigma) for 30 min, washed, then imaged. For denaturing immunoblots, samples were electrophoresed on Criterion 4 to 20% TGX Stain-Free Gels under denaturing, reducing conditions and imaged for protein. Gels were transferred to PVDF membranes (Fisher) overnight using Criterion Blotter (BioRad), then blocked for 1 h in 5% nonfat skim milk/TBST. Blots were incubated with anti-Ftl1 (Proteintech, #10727-1-AP) or anti-Fth1 (Cell Signaling, #3998S) at 1:1000 or with anti-GAPDH (Proteintech, #10494-1-AP) at 1:5000 for 2 h, washed with TBST for 5 min four times, incubated with anti-rabbit HRP-linked IgG (Proteintech, #SA00001-2) at 1:5000 for 1 h, washed, then imaged using Amersham ECL Prime (Cytiva) and a ChemiDoc. For native immunoblots, samples were electrophoresed under native conditions and transferred to PVDF; blots were UV-crosslinked, boiled in 50 mM Tris pH 6.8/1% SDS/2% β-mercaptoethanol for 10 min, washed with water, then blocked and probed as above. For methanol/heat treatments, samples were treated with 40% methanol, heated at 50 °C for 10 min, and centrifuged at 14,000*g* for 15 min at 4 °C. Pellets were resuspended in 8 M urea for electrophoresis or resuspended in acid for metal analysis. Supernatants were centrifuged in Ultra-0.5 centrifugal filter units with 100 kD molecular weight cutoffs (Millipore Sigma) at 14,000*g* for 30 min at 4 °C.

### Statistical analysis

Statistics were performed using GraphPad Prism 8 except for mass spectrometry data. Data were tested for normal distribution by Shapiro–Wilk test; if not normally distributed, data were log transformed. Groups within each sex were compared by one-way ANOVA with Tukey’s multiple comparisons test or by unpaired, two-tailed *t* test as indicated in figure legends. *p* < 0.05 was considered significant. Data are represented as means ± standard deviation.

## Data availability

Raw mass spectrometry data are available at University of California San Diego MassIVE (https://massive.ucsd.edu/ProteoSAFe/dataset.jsp?task=b2f5db6169b446c4a1ab2fb152493c44). All other data are contained within the article.

## Supporting information

This article contains supporting information.

## Conflict of interest

The authors declare that they have no conflicts of interest with the contents of this article.
