# Supplementary material for: Biliary excretion of excess iron in mice requires hepatocyte iron import by Slc39a14
Source: J Biol Chem. 2021 May 26;297(1):100835. doi: 10.1016/j.jbc.2021.100835 (PMC8214222; doi:10.1016/j.jbc.2021.100835)
Supplement: Supplemental Figures S1–S7 and Table S1 [file mmc2.pdf]

Supplementary Information

Biliary excretion of excess iron in mice requires hepatocyte iron import by Slc39a14

Milankumar Prajapati, Heather L. Conboy, Shintaro Hojyo, Toshiyuki Fukada, Bogdan Budnik, Thomas B. Bartnikas

Contents:
Table S1. Details of calculations
Figure S1. Slc39a14 deficiency impairs liver Fe loading in mice raised on an Fe-rich diet.
Figure S2. Slc39a14 deficiency impairs liver Fe loading in mice raised on an Fe-rich diet.
Figure S3. Slc39a14 deficiency impairs biliary excretion of Fe-rich ferritin in mice on Fe–rich diet.
Figure S4. Slc39a14 deficiency results in decreased levels of holo-ferritin in bile.
Figure S5. Methanol/heat treatment enriches for ferritin in liver lysates but not bile.
Figure S6. Slc30a10 deficiency does not impair Fe loading in mice raised on an Fe-rich diet.
Figure S7. Slc30a10 deficiency does not impair biliary excretion of excess Fe.

Table S1. Details of calculations.

Calculation	Details
How much Fe can bile Tf bind? (Slc39a14)	• 50-100 μg Tf/mL bile (Fig. 2B)<br>• Molecular weight Tf: 80 kDa<br>• 50-100 ug Tf/mL bile = 0.05-0.1 g Tf/L bile<br>• 0.05-0.1 g Tf/L bile / 80 000 g/mol = 0.625-1.25 μM<br>• Tf can bind two Fe atoms<br>• 0.625-1.25 μM Tf can bind 1.25-2.5 μM Fe<br>• Molecular weight Fe: 55.847 Da<br>• 1.25-2.5 μM Fe = 1.25-2.5 x 10^{-6} mol/L<br>• 1.25-2.5 x 10^{-6} mol Fe/L x 55.847 g Fe/mol Fe = 69.8-139.6 μg Fe/L = 69.8-139.6 ng Fe/mL bile
How much Fe can bile ferritin bind? (Slc39a14)	• 20 μg non-heme Fe/mL bile (Fig. 1B)<br>• Molecular weight Fe: 55.847 Da<br>• 20 μg non-heme Fe/mL bile = 0.02 g Fe/L bile<br>• 0.02 g Fe/L bile / 55.847 g/mol = 358.1 μM<br><br>• 175 μg Ftl1/mL bile (Fig. 2D)<br>• Molecular weight Ftl1: 21 kDa<br>• 175 μg Ftl1/mL bile = 0.175 g Ftl1/L bile<br>• 0.175 g Ftl1/L bile / 21000 g/mol = 8.3 μM<br><br>• Assuming 24 subunits per ferritin and 8.3 μM Ftl1, ferritin concentration = 8.3 μM / 24 = 0.35 μM ferritin<br>• Ratio of non-heme Fe to ferritin = 358.1 μM non-heme Fe / 0.35 μM ferritin = 1031
How much heme Fe is RBC-derived? (Slc39a14)	• 40.4 ug Hba/mL bile<br>• Molecular weight Hba: 15 kDa<br>• 40.4 ug Hba/mL bile = 0.0404 g Hba/L bile<br>• 0.0404 g Hba/L bile / 15000 g/mol = 2.7 μM

	• Assuming each hemoglobin contains 2 Hba and 4 Fe…<br>• Bile contained 2.7 uM x 2 = 5.4 μM RBC-derived heme Fe

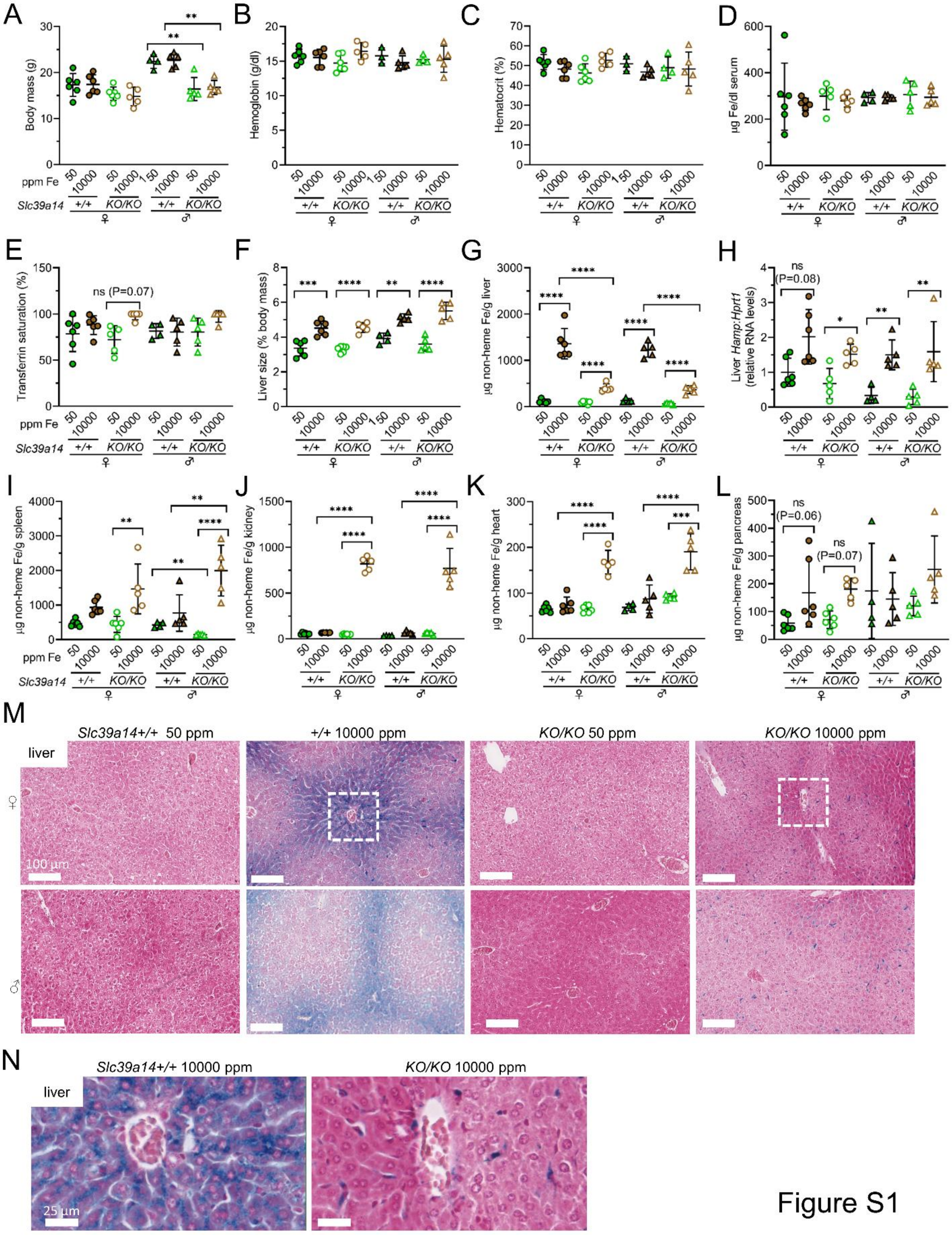
A
B
C
D
E
F
G
H
I
J
K
L
M
N
Body mass (g)
Hemoglobin (g/dl)
Hematocrit (%)
µg Fe/dl serum
Transferrin saturation (%)
Liver size (% body mass)
µg non-heme Fe/g liver
Liver Hamp:Hprt1 (relative RNA levels)
µg non-heme Fe/g spleen
µg non-heme Fe/g kidney
µg non-heme Fe/g heart
µg non-heme Fe/g pancreas
ppm Fe
Slc39a14
+/+
KO/KO
ns (P=0.07)
ns (P=0.08)
ns (P=0.06)
Slc39a14+/+ 50 ppm
+/+ 10000 ppm
KO/KO 50 ppm
KO/KO 10000 ppm
liver
100 µm
Slc39a14+/+ 10000 ppm
KO/KO 10000 ppm
25 µm

Figure S1

Figure S1. Slc39a14 deficiency impairs liver Fe loading in mice raised on an Fe-rich diet. *Slc39a14*$^{+/+}$ and *Slc39a14*$^{KO/KO}$ mice were weaned onto Fe-sufficient (50 ppm) or –rich (10,000 ppm) diets, then underwent collection of bile, blood, and tissues at two months of age. (A) Body mass. (B) Hemoglobin levels. (C) Hematocrits. (D) Serum Fe levels. (E) Transferrin saturations. (F) Liver mass as percent of body mass. (G) Liver non-heme Fe levels. (H) Liver hepcidin (*Hamp*) RNA levels relative to *Hprt1* RNA levels, with values in each group normalized to average value in female *Slc39a14*$^{+/+}$ mice on 50 ppm diet. (I-L) Non-heme Fe levels in spleen (I), kidney (J), heart (K), and pancreas (L). Bars indicate mean +/- standard deviation. Groups within each sex were compared by one-way ANOVA with Tukey's post-hoc test (ns P=>0.05, * P<0.05, ** P<0.01, *** P<0.001, **** P<0.0001). (M, N) Liver Fe stains at 10x (M) and 40x (N); scale bar indicates 100 μm (M) or 25 μm (N). Images in (N) correspond to dashed boxes in (M).

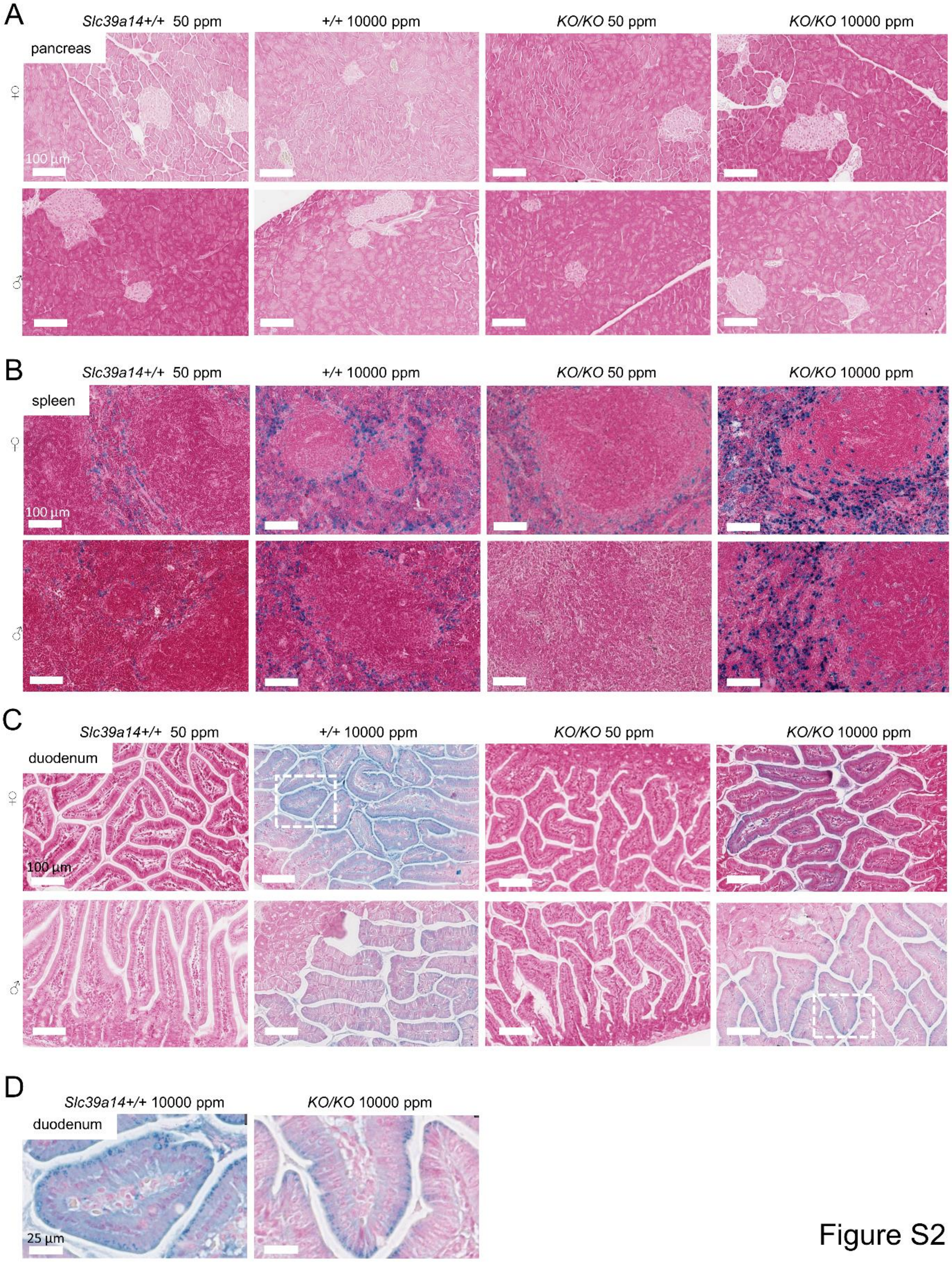
A
Slc39a14+/+ 50 ppm
+/+ 10000 ppm
KO/KO 50 ppm
KO/KO 10000 ppm
pancreas
♀
♂
100 µm
B
Slc39a14+/+ 50 ppm
+/+ 10000 ppm
KO/KO 50 ppm
KO/KO 10000 ppm
spleen
♀
♂
100 µm
C
Slc39a14+/+ 50 ppm
+/+ 10000 ppm
KO/KO 50 ppm
KO/KO 10000 ppm
duodenum
♀
♂
100 µm
D
Slc39a14+/+ 10000 ppm
KO/KO 10000 ppm
duodenum
25 µm

Figure S2

Figure S2. Slc39a14 deficiency impairs liver Fe loading in mice raised on an Fe-rich diet.
Slc39a14$^{+/+}$ and *Slc39a14*$^{KO/KO}$ mice were weaned onto Fe-sufficient or –rich diets, then underwent collection of bile, blood, and tissues at two months of age. Pancreas (A), spleen (B), and small intestine (C, D) Fe stains at 10x (A-C) or 40x (D); scale bar indicates 100 μm (A-C) or 25 μm (D). Images in (D) correspond to dashed boxes in (C).

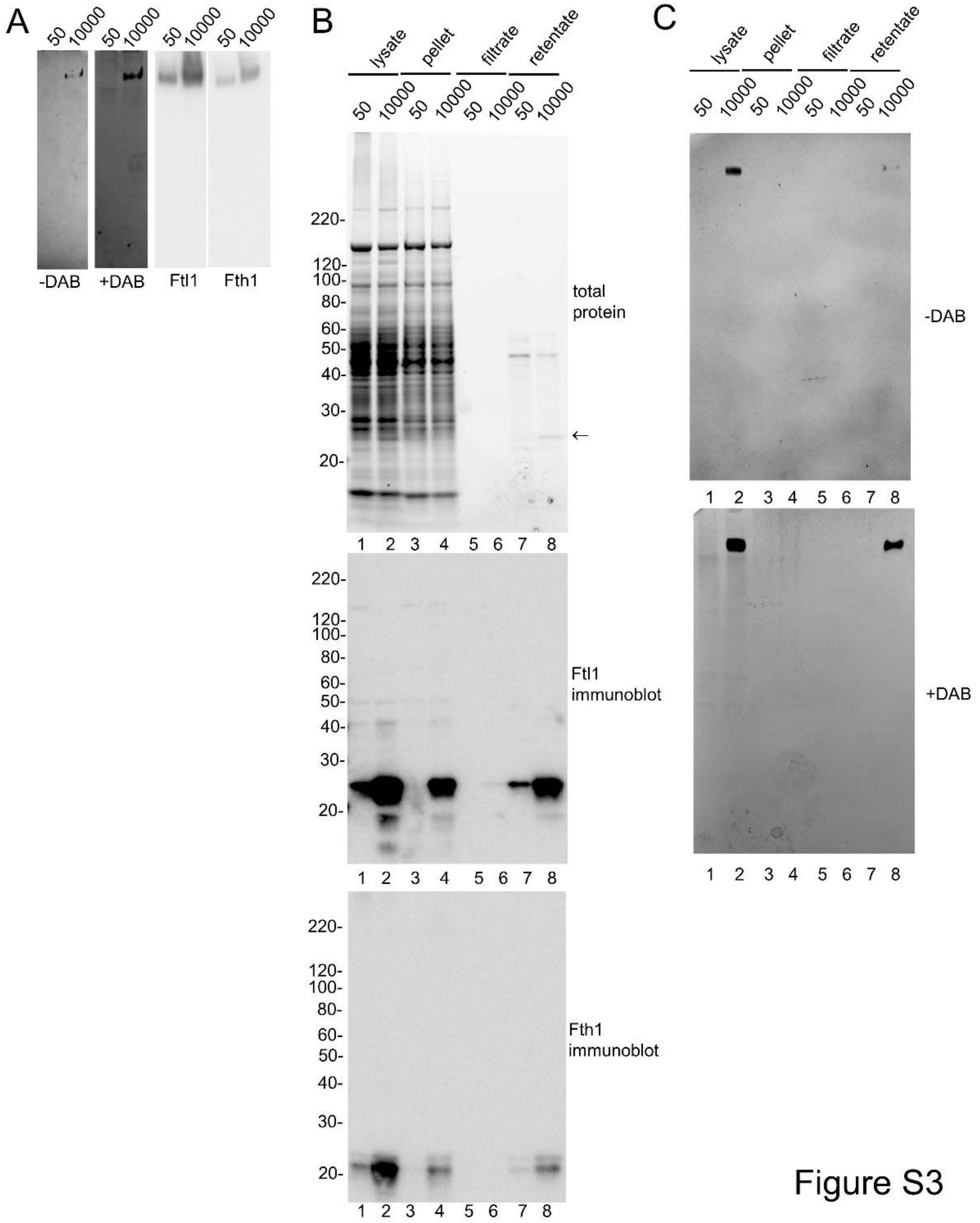

Figure S3. Slc39a14 deficiency impairs biliary excretion of Fe-rich ferritin in mice on Fe–rich diet. (A) 25 ug liver lysates from male *Slc39a14*$^{+/+}$ mouse on Fe-sufficient or -rich diet were electrophoresed under native conditions and subjected to Fe staining, without (-DAB) and with (+DAB) enhancement (left two blots), or immunoblotting with anti-Ftl1 and Fth1 antibodies (right two blots). (B, C) 50-100 mg livers from 4-5 male *Slc39a14*$^{+/+}$ on Fe-sufficient or -rich diets were pooled and homogenized. 2.5 mg liver lysates were subjected to methanol and heat treatment and centrifugal filtration. 50 ug liver lysates and 1/50 total volume of pellet, filtrate, and retentates were electrophoresed under denaturing conditions and visualized for protein or immunoblotted for Ftl1 or Fth1 (top three images; arrow indicates differentially expressed protein co-migrating with Ftl1) (B). Samples were electrophoresed under native conditions and subjected to Fe staining without and with enhancement (bottom two images) (C).

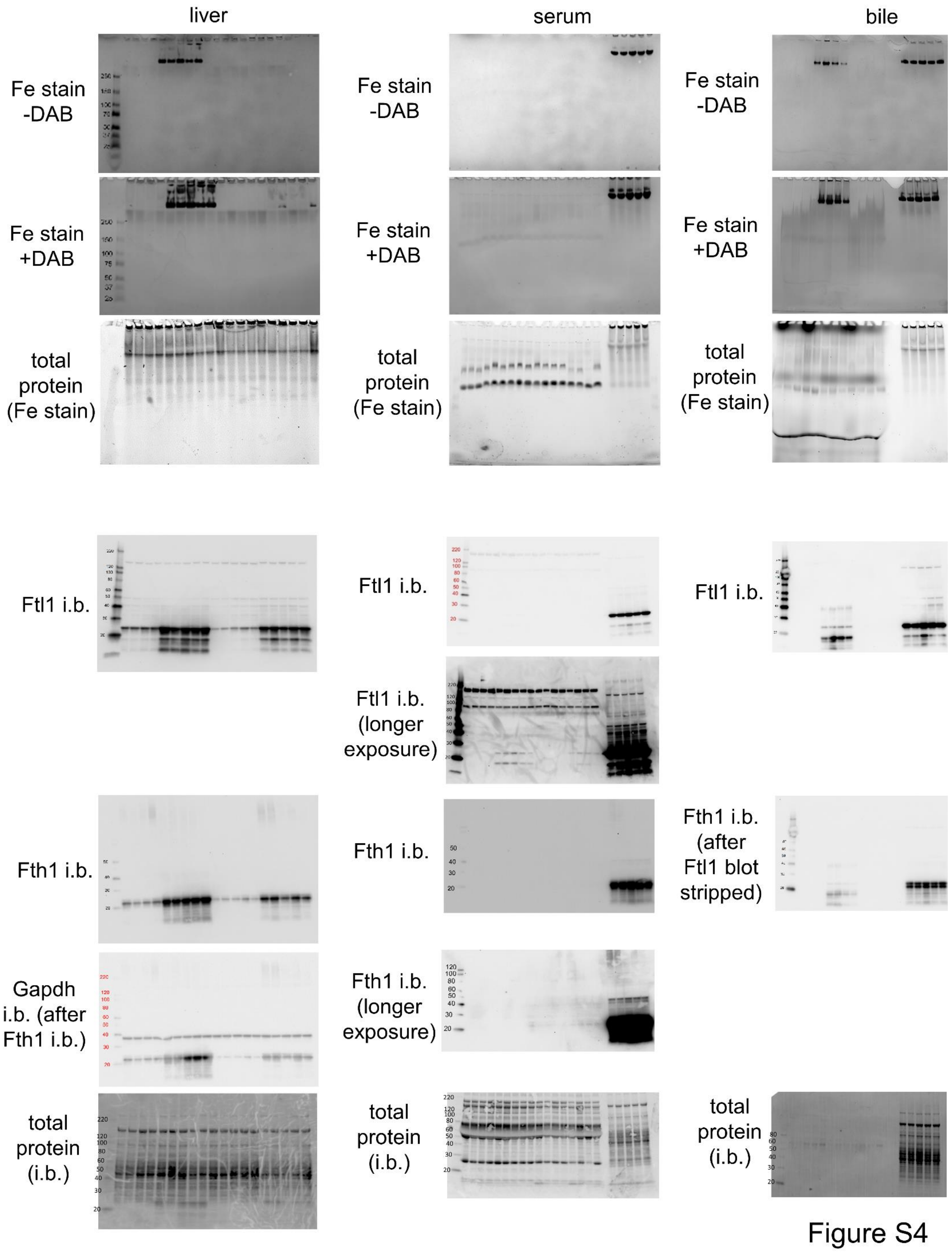

Figure S4. Slc39a14 deficiency results in decreased levels of holo-ferritin in bile. Uncropped gel images and blots from Fig. 3A-C.

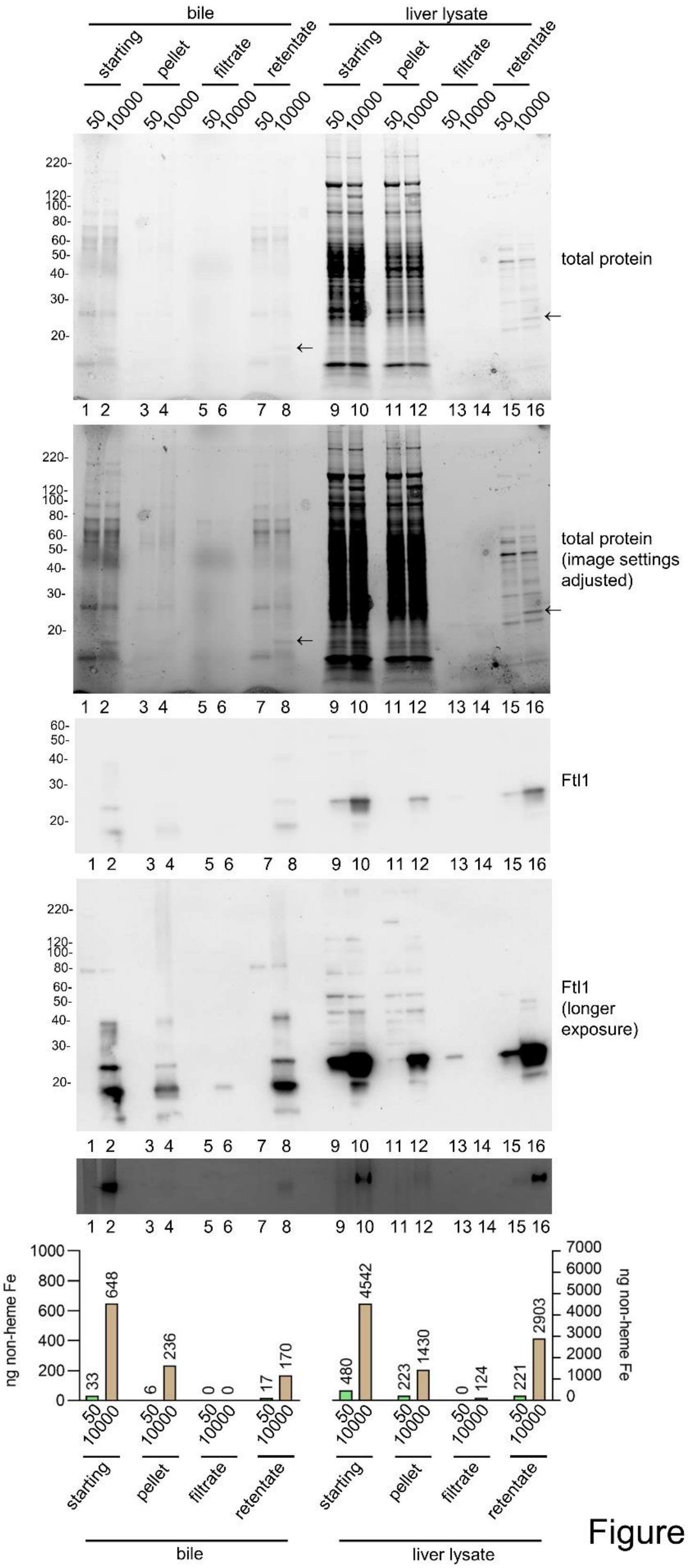
bile
liver lysate
starting
pellet
filtrate
retentate
50
10000
220-
120-
100-
80-
60-
50-
40-
30-
20-
total protein
1 2 3 4 5 6 7 8 9 10 11 12 13 14 15 16
total protein (image settings adjusted)
Ftl1
Ftl1 (longer exposure)
ng non-heme Fe
33
648
6
236
0
0
17
170
480
4542
223
1430
0
124
221
2903

Figure S5

Figure S5. Methanol/heat treatment enriches for ferritin in liver lysates but not bile. Bile samples from 4-5 male *Slc39a14*$^{+/+}$ on Fe-sufficient or -rich diets were pooled. Pooled liver lysates from Fig. S3B, C were analyzed in parallel. 20 ul bile and 2.5 mg liver lysates were subjected to methanol/heat treatment and centrifugal filtration. 1 ul bile, 1/20 of bile factions, 50 ug of liver lysates, and 1/50 of liver lysates fractions were electrophoresed under denaturing conditions and visualized for total protein (top two blots; bottom image adjusted to better visualize differentially expressed band in retentates, as indicated by arrow). Samples were also immunoblotted under denaturing conditions with anti-Ftl1 (third and fourth images from the top, with lower image representing a longer exposure) and under native conditions and subjected to Fe staining with enhancement (bottom image). Samples were also analyzed for non-heme Fe levels; Fe levels in entire starting sample or fraction are shown in bottom graph.

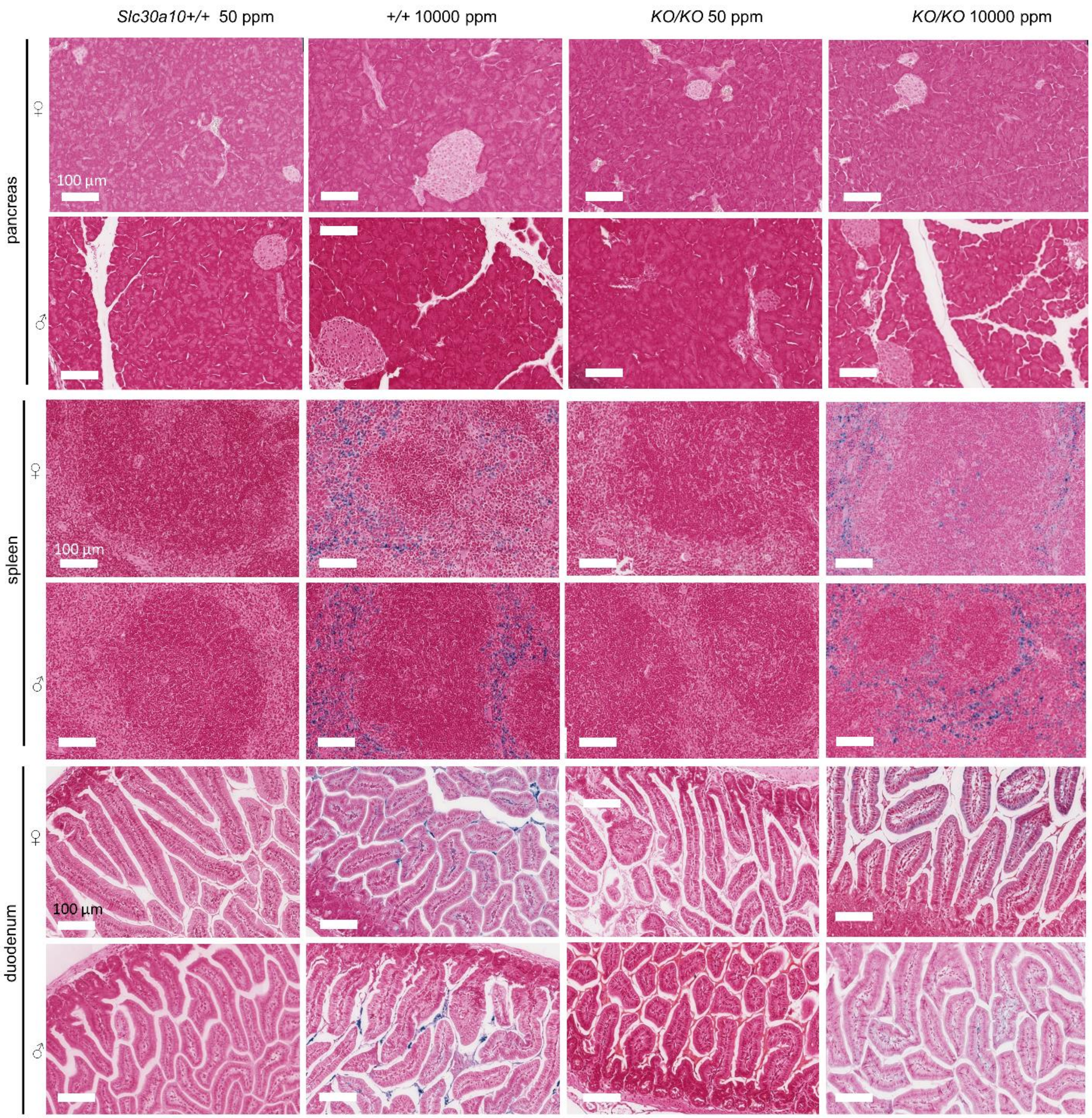

Figure S6

Figure S6. Slc30a10 deficiency does not impair Fe loading in mice raised on an Fe-rich diet. *Slc30a10*$^{+/+}$ and *Slc30a10*$^{KO/KO}$ mice were weaned onto Fe-sufficient or –rich diets, then underwent collection of bile, blood, and tissues at two months of age. Pancreas, spleen, and small intestine Fe stains at 10x; scale bar indicates 100 µm.

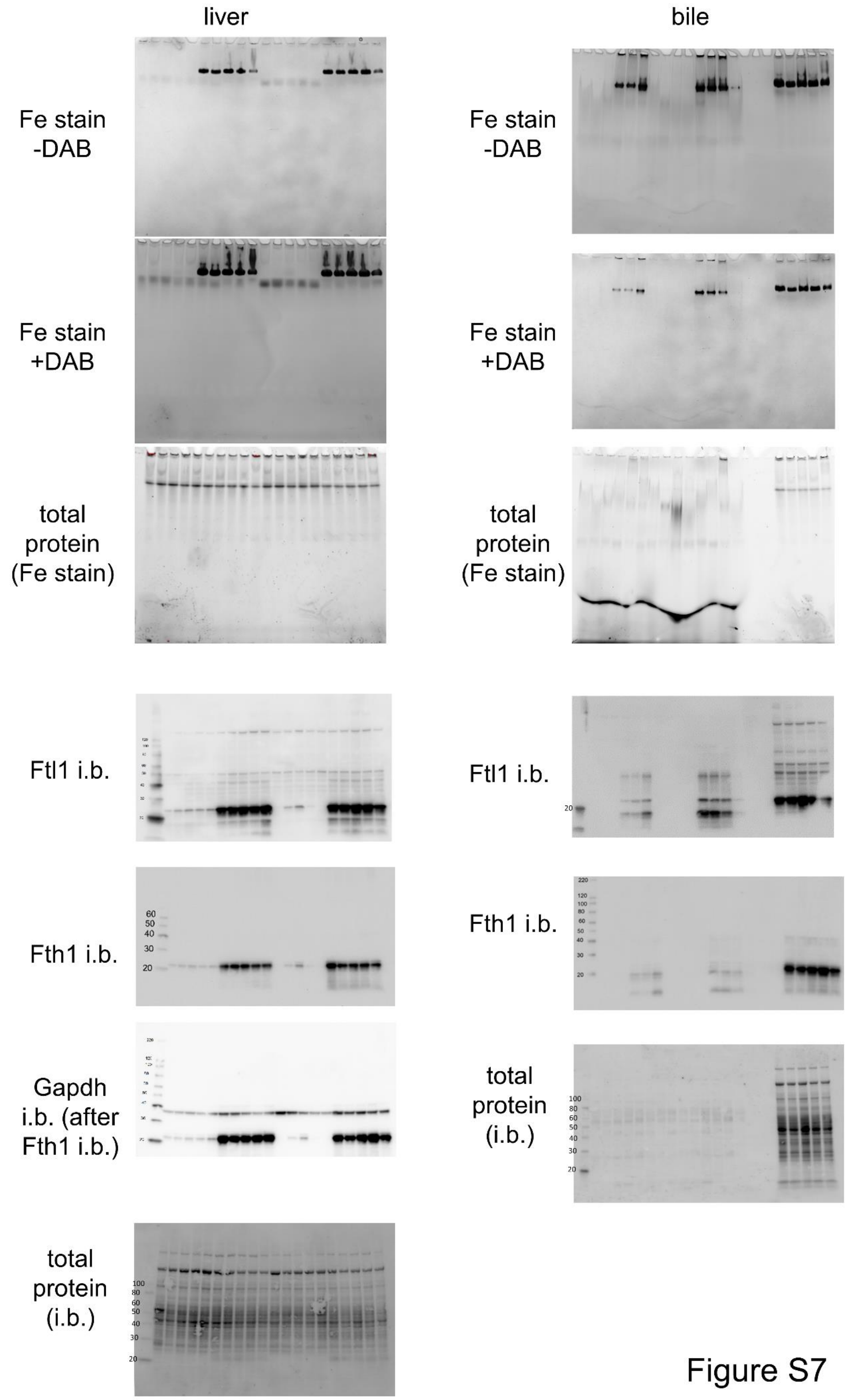

Figure S7. Slc30a10 deficiency does not impair biliary excretion of excess Fe. Uncropped gel images and blots from Fig. 5I, J.
